# CRISPRi-mediated functional analysis of NKX2-1-binding sites in the lung

**DOI:** 10.1038/s42003-021-02083-4

**Published:** 2021-05-12

**Authors:** William D. Stuart, Iris M. Fink-Baldauf, Koichi Tomoshige, Minzhe Guo, Yutaka Maeda

**Affiliations:** 1 Perinatal Institute, Division of Neonatology, Perinatal and Pulmonary Biology, Cincinnati Children’s Hospital Medical Center and the University of Cincinnati College of Medicine (CCHMC and UC), Cincinnati, OH USA; 2 Present Address: Department of Surgical Oncology, Nagasaki University Graduate School of Biomedical Sciences, Nagasaki, Japan

**Keywords:** Transcriptional regulatory elements, CRISPR-Cas9 genome editing, Respiratory tract diseases

## Abstract

The transcription factor NKX2-1/TTF-1 is involved in lung pathophysiology, including breathing, innate defense and tumorigenesis. To understand the mechanism by which NKX2-1 regulates genes involved in such pathophysiology, we have previously performed ChIP-seq and identified genome-wide NKX2-1-binding sites, which revealed that NKX2-1 binds to not only proximal promoter regions but also multiple intra- and inter-genic regions of the genes regulated by NKX2-1. However, the roles of such regions, especially non-proximal ones, bound by NKX2-1 have not yet been determined. Here, using CRISPRi (CRISPR/dCas9-KRAB), we scrutinize the functional roles of 19 regions/sites bound by NKX2-1, which are located in genes involved in breathing and innate defense (*SFTPB, LAMP3*, *SFTPA1, SFTPA2*) and lung tumorigenesis (*MYBPH, LMO3, CD274/PD-L1*). Notably, the CRISPRi approach reveals that a portion of NKX2-1-binding sites are functionally indispensable while the rest are dispensable for the expression of the genes, indicating that functional roles of NKX2-1-binding sites are unequally yoked.

## Abstract (web summary)

The transcription factor NKX2-1 is a key regulator of lung pathophysiology, but the importance of its binding sites outside of proximal promoter regions is unclear. Here, William Stuart and Iris Fink-Baldauf et al. use CRISPRi to interrogate 19 NKX2-1 binding sites and identify specific sites important for breathing, innate immune defense, and tumorigenesis.

## Introduction

Ever since it was first shown that *Nkx2-1* (also known as TTF-1) deleted mice had impaired lung development, which results in death at birth due to a breathing defect^1^, the molecular mechanisms by which NKX2-1 regulates genes involved in lung development and physiology have been extensively pursued^2^. One of the genes critical for breathing at birth is *SFTPB* (also known as surfactant protein B gene), since *Sftpb*-deleted mice also succumb to death at birth because of a breathing defect due to collapsed lungs^3^. Using gel-shift (also known as EMSA [Electrophoretic mobility shift assay]) and plasmid-based reporter assays, NKX2-1 has been shown to bind to the proximal promoter region of human *SFTPB* and stimulate its promoter activity^4,5^. A microarray analysis to determine the genome-wide NKX2-1 downstream target genes in human lung epithelial cells also identified *SFTPB* as well as *LAMP3* (also known as Lysosomal Associated Membrane Protein 3), both of which co-localize at lamellar bodies (a cellular organelle) that process and secrete surfactant to reduce lung surface tension, which is required for proper lung function^6^. These studies indicate that NKX2-1 is indispensable for lung development involved in breathing in part through the regulation of *SFTPB* and *LAMP3* expression.

The microarray analysis also identified that *SFTPA* (also known as surfactant protein A gene), including *SFTPA1* and *SFTPA2* (a paralog of *SFTPA1*), is induced by NKX2-1 in human lung epithelial cells^6^, in part through the proximal promoters of *SFTPA1* and *SFTPA2* bound by NKX2-1, which has been reported using gel-shift and plasmid-based reporter assays^7–9^. Although SFTPA1/SFTPA2 belong to the same surfactant protein gene family as SFTPB, *Sftpa1*-deleted mice (of note, no *Sftpa2* in mouse) are viable at birth and have no apparent lung abnormality^10^. However, *Sftpa1*-deleted mice are susceptible to bacterial, viral, and fungal infection^11–15^, indicating that NKX2-1 is involved in innate defense by the clearance of respiratory bacteria, virus, and fungus through SFTPA.

Development of a monoclonal NKX2-1 antibody (8G7G3/1) revealed that NKX2-1 is expressed in normal human lung epithelium as well as in human lung carcinomas, especially lung adenocarcinoma and small-cell lung carcinoma, but not colon or breast carcinoma^16^. Since then, the antibody has been used extensively in the clinic to distinguish primary lung adenocarcinomas from lung metastases^17,18^. Notably, NKX2-1 is not only highly expressed in primary human lung adenocarcinoma, but also its chromosomal locus (14q13.3) is highly amplified in ~10% of human lung adenocarcinomas^19–22^. Discovery of the 14q13.3 amplification led to subsequent studies to understand the functional roles of NKX2-1 and its downstream target genes that influence lung tumorigenesis. Importantly, such studies have revealed that NKX2-1 functions as a tumor promoter or a tumor suppressor in a context-dependent fashion. For example, NKX2-1 promotes *EGFR*-mutant lung tumorigenesis while it suppresses *KRAS*-mutant lung tumorigenesis^23–29^. Among the downstream target genes induced by NKX2-1, *MYBPH*, *LMO3*, and *CD274* (also known as PD-L1) are of particular interest since they directly influence lung tumorigenesis^24,28,30^. MYBPH (also known as myosin binding protein H) inhibits cell motility and metastasis of lung adenocarcinoma^24^ while LMO3 promotes cell survival^28^. CD274/PD-L1, a suppressor of adaptive immunity, expressed on lung tumor cells binds to PD-1 on T cells and inhibits the anti-tumorigenic activity of T cells^31^. These reports indicate that NKX2-1 influences lung tumorigenesis in part through the regulation of *MYBPH*, *LMO3,* and *CD274/PD-L1* expression.

**Fig. 1:**
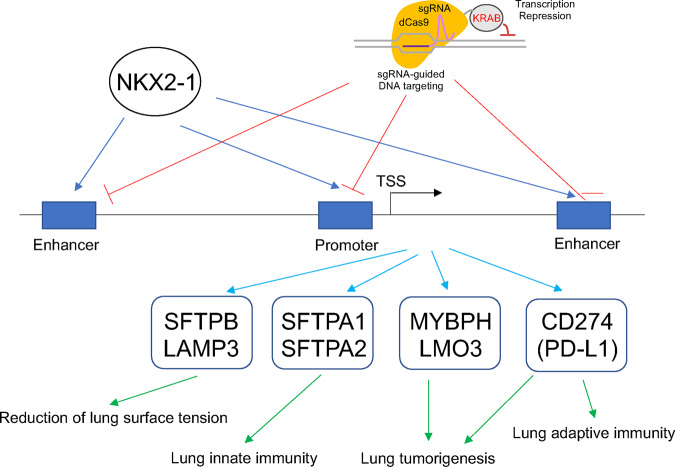
Experimental scheme of CRISPRi analysis assessing the roles of NKX2-1-binding sites. NKX2-1-binding sites identified by ChIP-seq were targeted by CRISPRi that carries dCas9-KRAB and sgRNAs, which match to each binding site sequence, and the expression of NKX2-1 downstream target genes (*SFTPB*, *LAMP3*, *SFTPA1*, *SFTPA2*, *MYBPH*, *LMO3*, and *CD274/PD-L1*) was assessed. The putative lung function of each gene is shown. TSS transcription start site.

The regulatory mechanisms by which NKX2-1 activates expression of these genes had previously been investigated by focusing on the proximal promoter (upstream) regions using gel-shift and plasmid-based reporter assays, which do not provide a chromatin-based and genome-wide mechanism of gene regulation. Subsequently, we used the ChIP (chromatin immunoprecipitation)-seq assay to determine unbiased genome-wide NKX2-1 binding sites in human lung epithelial cells^27^. ChIP-seq analysis by us and others indicated that NKX2-1 bound to not only proximal upstream regions but also distal upstream and/or downstream regions as well as intronic regions and intergenic regions in the genome^27–29,32^. Although ChIP-seq can reveal where NKX2-1 binds in the genome, it does not indicate whether NKX2-1-binding sites are functionally important or not. The ability to determine the functional relevance of transcription factor-binding sites has recently been achieved using CRISPR/Cas9 technology. Mansour and colleagues using a CRISPR/Cas9-mediated deletion approach, deleted a transcription factor MYB-binding site (identified by ChIP-seq) between *TAL1* and *STIL*, and showed that the MYB-binding site is required for the expression of *TAL1*^33^. We have previously used the same approach and determined that a transcription factor SPDEF-binding site (identified by ChIP-seq) between *MUC5AC* and *MUC5B* is required for the expression of *MUC5B*^30^. We have also recently used a modified CRISPR/Cas9 technology (CRISPRi) that carries a deactivated Cas9 fused to the repressor KRAB (CRISPR/dCas9-KRAB) to repress the transcription of genes without creating DNA double-stranded breaks. Gene-regulatory regions in the genome that are targeted by CRISPRi are selected by 20-mer sgRNA target sequences^34,35^. We thus targeted the SPDEF-binding region and found that the CRISPRi approach is more streamlined than the CRISPR/Cas9-mediated deletion approach since cell cloning is not required in CRISPRi^36^. In the present study, using the CRISPRi approach, we set out to determine whether NKX2-1-binding sites that were previously identified by ChIP-seq are indispensable for the expression of NKX2-1 downstream target genes, including *SFTPB, LAMP3, SFTPA1, SFTPA2, MYBPH, LMO3*, and *CD274/PD-L1*, that are involved in breathing, innate defense, and tumorigenesis (Fig. 1).

## Results

### First intron of *SFTPB* bound by NKX2-1 is most critical for the expression of *SFTPB* induced by NKX2-1

**Fig. 2:**
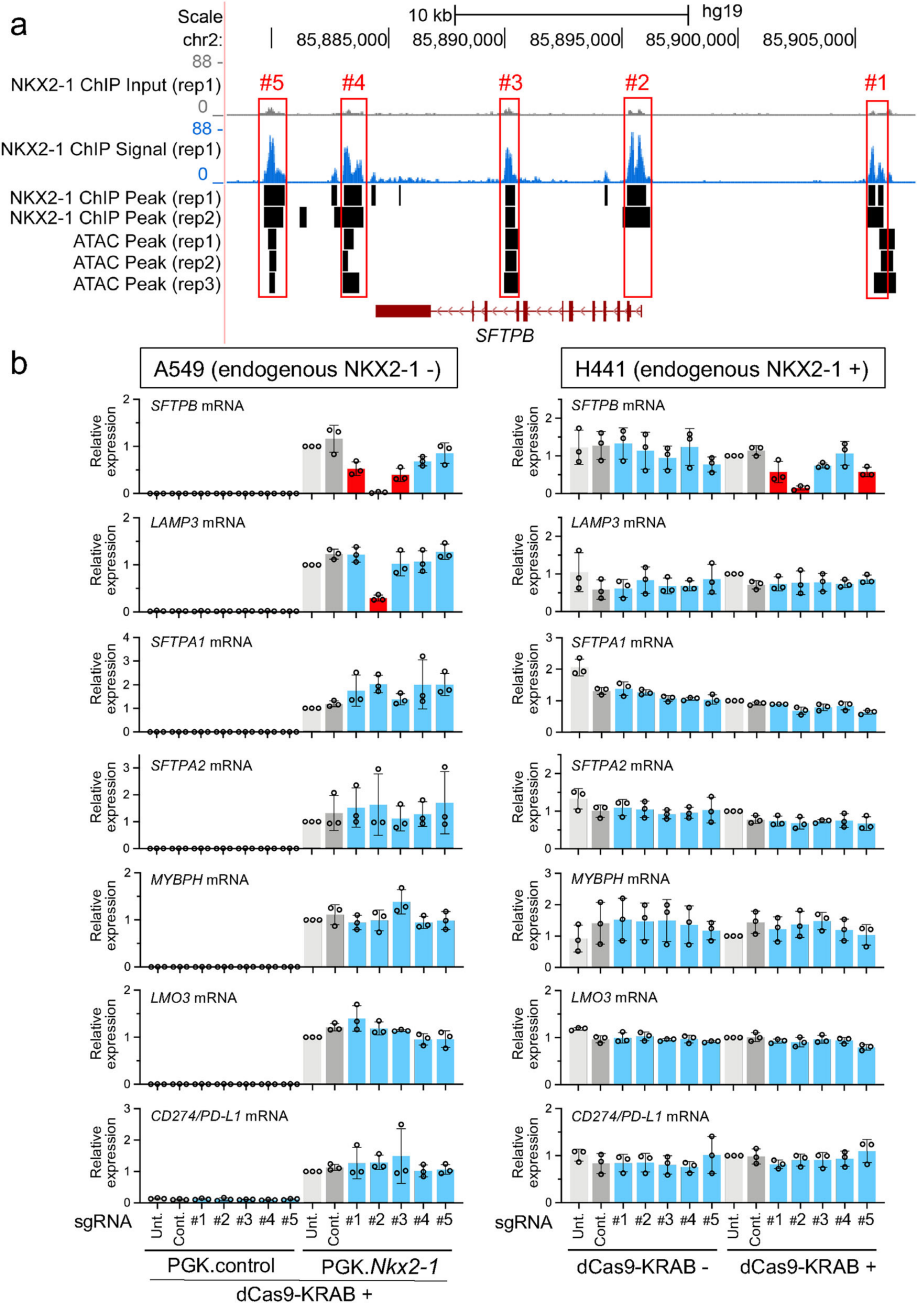
NKX2-1-binding sites located at the upstream and first intronic regions are commonly required for the expression of *SFTPB* induced by NKX2-1. **a** Shown are ATAC-seq and ChIP-seq data indicating that NKX2-1 binds to upstream (#1), first (#2), ninth intronic (#3), proximal (#4), and distal downstream (#5) regions of human *SFTPB* locus. ChIP-seq was previously performed using an A549 lung epithelial cell line stably expressing NKX2-1^27,30^. ATAC-seq was obtained from the ENCODE database^37^. NKX2-1-binding sites are shown as peaks obtained from bam files. Significant peaks compared to input peaks are shown as side bars obtained from bed files. **b** Shown is the gene expression of NKX2-1 downstream target genes affected by CRISPRi from biologically independent triplicates, in which synthetic sgRNAs targeting NKX2-1-binding sites (#) located at the regions described in (**a**) are transiently transfected into A549 cells expressing dCas9-KRAB with or without NKX2-1 (PGK.*Nkx2-1* or PGK vector control), or H441 cells with or without dCas9-KRAB. The sgRNA targeting NKX2-1-binding sites located at the upstream (#1) and first intronic (#2) regions significantly suppressed the expression of *SFTPB* in both A549 and H441 cells. The sgRNA targeting NKX2-1-binding sites located at the first intronic (#2) region also significantly suppressed the expression of *LAMP3* induced by NKX2-1 in A549 cells. Of note, endogenous *NKX2-1* is expressed in H441 cells but not in A549 cells (see Supplementary Fig. 1). Results are expressed as the mean ± SD of the triplicates for each group. Only *P* < 0.05 and more than two-fold suppression are considered significant CRISPRi-mediated suppression (highlighted in red). Unt., untransfected. Cont., non-targeted control sgRNA.

**Fig. 3:**
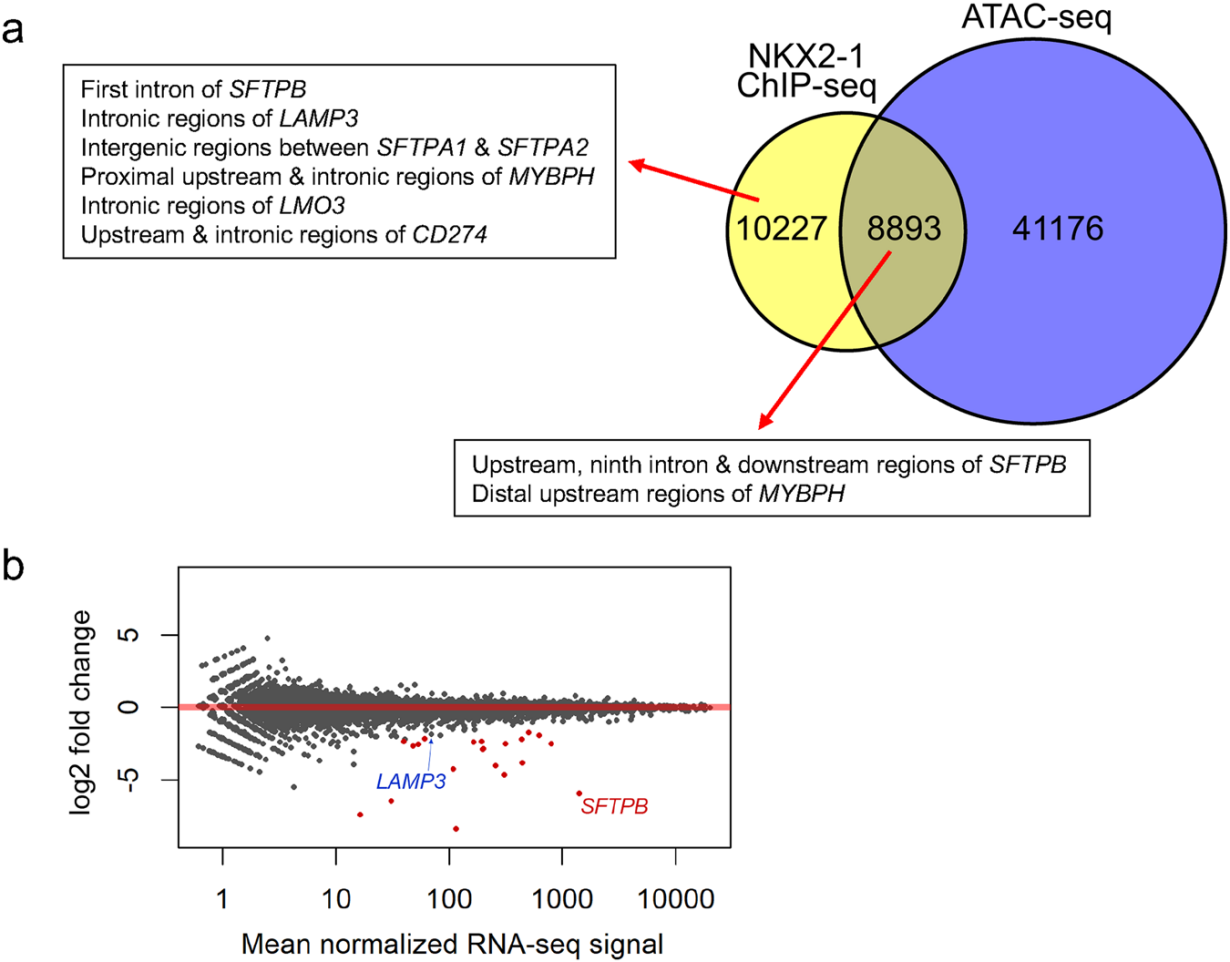
Genome-wide RNA-seq analysis indicates that the expression of *SFTPB* induced by NKX2-1 is most significantly repressed by CRISPRi targeting the first intron of *SFTPB* that is an ATAC-inaccessible region. **a** Shown are pie charts that indicate the number of NKX2-1 ChIP-seq peaks and ATAC-seq peaks in A549 cells. A portion (but not all) of the NKX2-1-binding sites are located in ATAC-accessible genomic regions. **b** Shown is an MA plot that indicates differential gene expression regulated by CRISPRi targeting the first intron (#2) of *SFTPB*, which was analyzed by RNA-seq using RNA from A549 cells that express dCas9-KRAB and NKX2-1 with sgRNA #2 or the sgRNA control as described in Fig. 2b. Biologically independent triplicates were used for each group (sgRNA #2 or sgRNA control). Red points represent genes with log2 fold change ≦1 or ≧−1; padj <0.05. The expression of *SFTPB* was most significantly repressed (log2 fold change −5.96; *p* value 2.06E−257; padj 3.06E−253) while the expression of *LAMP3* was weakly repressed (log2 fold change −1.85; *p* value 2.64E−03; padj 1.00E + 00).

Our previous ChIP-seq data from an A549 human lung epithelial cell line stably expressing NKX2-1^27,30^ indicate that NKX2-1 binds to five regions (upstream, first intron, ninth intron, proximal downstream, distal downstream) of the locus of *SFTPB*, which is an indispensable gene for breathing (Fig. 2a). Notably, ATAC-seq (Assay for Transposase-Accessible Chromatin using sequencing) data from A549 cells obtained from ENCODE^37^ indicated that an ATAC peak was not observed at an NKX2-1-binding site in the first intronic region, suggesting that NKX2-1 is able to access genomic regions that are considered to be inaccessible by ATAC-seq (Figs. 2a, 3a, and Supplementary Data 1). In order to understand which NKX2-1-binding sites in the five regions are important for the expression of *SFTPB* induced by NKX2-1, we created A549 cells stably expressing dCas9-KRAB with or without NKX2-1 (Supplementary Figs. 1 and 2) and transfected synthetic sgRNAs targeting individual DNA elements within or near NKX2-1-binding motifs (CTTG/CAAG) in the five regions of *SFTPB* (Fig. 2a and Supplementary Fig. 3). Of note, A549 cells do not express endogenous NKX2-1 (Supplementary Figs. 1 and 4). We then assessed the expression of *SFTPB, LAMP3, SFTPA1, SFTPA2, MYBPH, LMO3*, and *CD274/PD-L1*. This is a CRISPRi approach that we previously reported^36^. Notably, sgRNAs targeting the upstream region and first and ninth introns of the *SFTPB* locus significantly suppressed the expression of *SFTPB* more than two-fold (*P* < 0.05, red), indicating that these three regions (upstream, first, and ninth introns) are critical gene-regulatory regions for the expression of *SFTPB* induced by NKX2-1. The sgRNA targeting the first intronic region most extensively suppressed the expression of *SFTPB* compared to those targeting the upstream region and ninth intron (98.0% vs. 54.8 and 66.0%; Fig. 2b). The specificity of CRISPRi targeting this region was further confirmed by genome-wide RNA-seq (Fig. 3b and Supplementary Data 2). The sgRNAs targeting the proximal and distal downstream regions of *SFTPB* did not suppress the expression of *SFTPB*. Unexpectedly, the sgRNA targeting the first intronic region also suppressed the expression of *LAMP3* more than two-fold (*P* < 0.05) (Fig. 2b) though, according to the CRISPOR off-target search^38^ (Supplementary Data 3), the sgRNA sequence does not match a genomic DNA sequence at the *LAMP3* locus. Since the siRNA-mediated inhibition of endogenous *SFTPB* did not affect the expression of *LAMP3* (Supplementary Fig. 5), the first intronic region may regulate the expression of *LAMP3* in *trans* (of note, *SFTPB* and *LAMP3* are located at chromosomes 2 and 3, respectively). Hi-C data from A549 cells obtained by ENCODE data^37^ indicate that the *SFTPB* locus on chromosome 2 interacts with other loci in different chromosomes, including chromosome 3, supporting a possibility of such *trans* inter-chromosomal interactions (Supplementary Fig. 6).

We also created an H441 human lung epithelial cell line that stably expresses dCas9-KRAB to complement the above study using A549 cells. Of note, H441 cells express endogenous *NKX2-1* while A549 cells do not (Supplementary Fig. 1). We assessed which NKX2-1 downstream target genes identified by a gain-of-function analysis using A549 cells^27^ were regulated by NKX2-1 in H441 cells by a loss-of-function approach using siRNA. The expression of *SFTPB*, *SFTPA1*, *SFTPA2*, *MYBPH*, and *LMO3* was reduced by two independent siRNAs targeting endogenous *NKX2-1*, while the expression of *LAMP3* and *CD274/PD-L1* was not, suggesting that the expression of *SFTPB*, *SFTPA1*, *SFTPA2*, *MYBPH*, and *LMO3* (but not that of *LAMP3* and *CD274/PD-L1*) is regulated by NKX2-1 in H441 cells (Supplementary Fig. 7). As described above, we then transfected H441 cells that express dCas9-KRAB with the same sgRNAs and assessed the expression levels of the respective genes (Fig. 2b). Notably, sgRNAs targeting the upstream and first intron of the *SFTPB* locus significantly suppressed the expression of *SFTPB* (Fig. 2b), which is consistent with the results using A549 cells (Fig. 2b); however, the sgRNA targeting the ninth intron of *SFTPB*, which was suppressive in A549 cells, was not suppressive in H441 cells, suggesting a cell type-specific gene regulation. Conversely, while sgRNA targeting the distal downstream region of *SFTPB* did not suppress the expression of *SFTPB* in A549 cells, expression was suppressed in H441 cells. These results suggest that the NKX2-1-binding sites located at the upstream and first intronic regions of *SFTPB* are essential to NKX2-1-mediated expression of *SFTPB* regardless of cell type; however, other regions function in a cell type-dependent fashion.

### Both the third and fifth introns of *LAMP3* bound by NKX2-1 are critical for the expression of *LAMP3* induced by NKX2-1

**Fig. 4:**
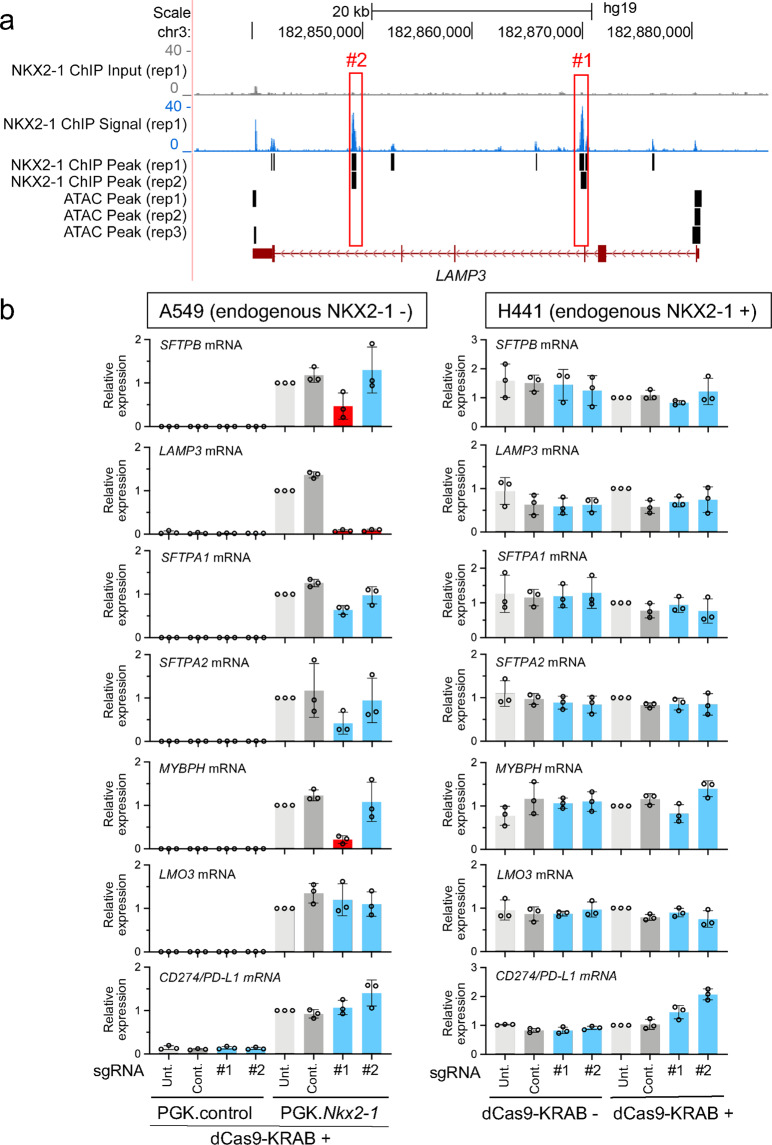
NKX2-1-binding sites located at the third and fifth intronic regions are required for the expression of *LAMP3* induced by NKX2-1 in A549 cells. **a** Shown are ATAC-seq and ChIP-seq data indicating that NKX2-1 binds to the third (#1) and fifth (#2) intronic regions of the human *LAMP3* locus. Significant peaks are described as in Fig. 2a. **b** Shown is gene expression of NKX2-1 downstream target genes affected by CRISPRi as described in Fig. 2b. The sgRNA targeting NKX2-1-binding sites located at the third (#1) and fifth (#2) regions significantly suppressed the expression of *LAMP3* induced by NKX2-1 in A549 cells but not in H441 cells. The sgRNA targeting NKX2-1-binding sites located at the third (#1) intronic region also significantly suppressed the expression of *SFTPB* and *MYBPH* induced by NKX2-1. Results are expressed as the mean ± SD of the triplicates for each group. Only *P* < 0.05 and more than twofold suppression are considered significant CRISPRi-mediated suppression (highlighted in red). Unt. untransfected, Cont. non-targeted control sgRNA.

The ChIP-seq data from an A549 lung epithelial cell line stably expressing NKX2-1^27,30^ also indicate that NKX2-1 binds to two regions (third and fifth introns) of the locus of *LAMP3*, a product of which associates with SFTPB (Fig. 4a). Notably, these two NKX2-1-binding sites are not in the ATAC-accessible regions (Fig. 4a), again suggesting that NKX2-1 is able to bind to genomic regions that are considered to be inaccessible by ATAC-seq (Fig. 3a and Supplementary Data 1). In order to understand which NKX2-1-binding sites in the two regions are important for the expression of *LAMP3* induced by NKX2-1, we took the same CRISPRi approach as described above except that sgRNAs targeting the two regions of *LAMP3* were used (Supplementary Fig. 3). Notably, both sgRNAs targeting their respective regions suppressed the expression of *LAMP3* more than twofold (*P* < 0.05) (Fig. 4b). Unexpectedly, the sgRNA targeting the third intronic region also suppressed the expression of *SFTPB* and *MYBPH* more than twofold (*P* < 0.05) (Fig. 4b) though they are located at different chromosomes from *LAMP3*. Of note, *LAMP3*, *SFTPB*, and *MYBPH* are located at chromosomes 3, 2, and 1, respectively. The CRISPOR off-target search^38^ does not indicate that the sgRNA sequence matches the genomic DNA sequences of the *SFTPB* and *MYBPH* loci (Supplementary Data 4), suggesting that the third intronic region may regulate the expression of *LAMP3* in *cis* and those of *SFTPB* and *MYBPH* in *trans*. Since the sgRNA targeting the fifth intronic region suppressed the expression of *LAMP3* at a similar level to the one targeting the third intronic region but did not suppress the expression of the other genes, the suppressed expression of *SFTPB* and *MYBPH* may not occur through the reduced expression of *LAMP3* mRNA and/or protein. As expected, these sgRNAs did not affect the expression of *LAMP3* or other NKX2-1 downstream target genes in H441 cells (Fig. 4b) since the siRNA-mediated loss-of-function experiment indicates that NKX2-1 does not largely regulate the expression of *LAMP3* in H441 cells (Supplementary Fig. 7), indicating a cell type-specific gene regulation by NKX2-1.

### The intergenic region between *SFTPA1* and *SFTPA2* carrying two NKX2-1-binding sites functions as a gene-regulatory region in *cis*

**Fig. 5:**
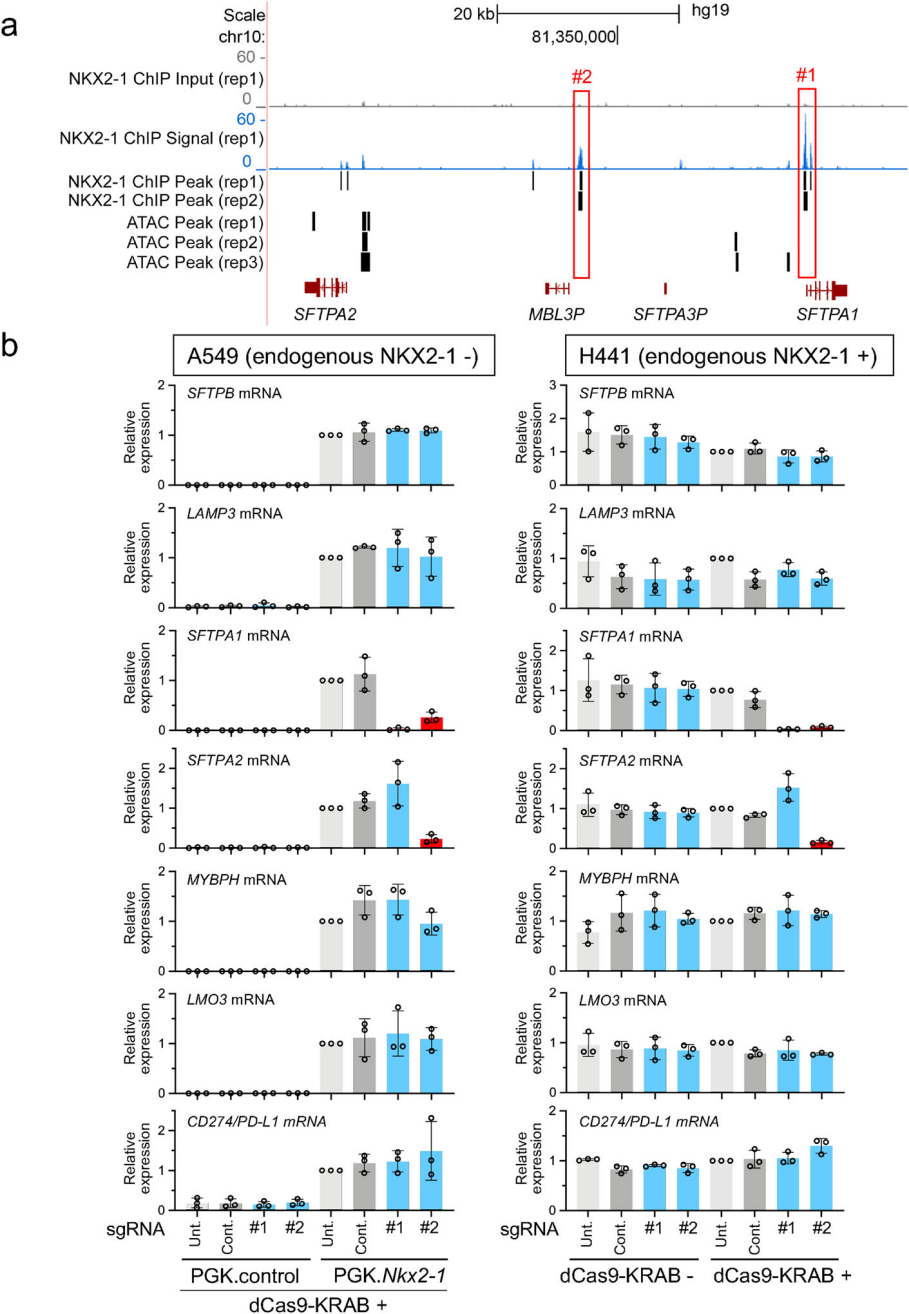
NKX2-1-binding sites located at two different regions between *SFTPA1* and *SFTPA2* function distinctively for the expression of *SFTPA1* and *SFTPA2* in both A549 and H441 cells. **a** Shown are ATAC-seq and ChIP-seq data indicating that NKX2-1 binds to an intergenic (#1 and #2) region of human *SFTPA1* and *SFTPA2*. Significant peaks are described as in Fig. 2a. *MBL3P* and *SFTPA3P* are pseudogenes. **b** Shown is gene expression of NKX2-1 downstream target genes affected by CRISPRi as described in Fig. 2b. The sgRNA targeting an NKX2-1-binding site located at the proximal upstream (#1) region of *SFTPA1* significantly suppressed the NKX2-1-induced expression of *SFTPA1* but not *SFTPA2*, while the one targeting an NKX2-1-binding site located at the distal upstream (#2) region of *SFTPA1* and *SFTPA2* significantly suppressed the NKX2-1-induced expression of both *SFTPA1* and *SFTPA2*. Results are expressed as the mean ± SD of the triplicates for each group. Only *P* < 0.05 and more than twofold suppression are considered significant CRISPRi-mediated suppression (highlighted in red). Unt. untransfected, Cont. non-targeted control sgRNA.

*SFTPA1* and its paralog *SFTPA2*, products of which are critical for innate defense against respiratory bacteria, viruses, and fungi, are located at chromosome 10q22.3. The ChIP-seq data^27,30^ indicate that NKX2-1 binds to a proximal upstream region of *SFTPA1* and a distal upstream region of *SFTPA1* and *SFTPA2*, which are located at ATAC-inaccessible regions (Fig. 5a and Supplementary Data 1). As described above, we performed the CRISPRi approach (Supplementary Fig. 3 and Supplementary Data 5) and assessed the gene-regulatory functions of these two regions in A549 cells. Notably, both sgRNAs targeting proximal and distal regions significantly suppressed the expression of *SFTPA1*; however, only the sgRNA targeting the distal region suppressed the expression of *SFTPA2* more than twofold (*P* < 0.05) (Fig. 5b). These sgRNAs did not affect the expression of other genes, including *SFTPB, LAMP3, MYBPH, LMO3*, and *CD274/PD-L1*, suggesting the two regions do not function in *trans* at least for these genes. Consistent with the results in A549 cells, the same pattern of gene regulation was observed in H441 cells (Fig. 5b).

Since NKX2-1 binds to a number of intragenic regions, we employed a CRISPRi approach that influences gene-regulatory activity without deleting a part of the genes. However, since the distal upstream region of *SFTPA1* and *SFTPA2*, which is bound by NKX2-1, is in an intergenic region (opposed to intragenic) that is ~20 kb away from both *SFTPA1* and *SFTPA2*, we also employed the CRISPR/Cas9-mediated approach to delete the distal upstream region and assessed the expression of *SFTPA1*, *SFTPA2*, and other NKX2-1 downstream target genes. Consistent with the results obtained by the CRISPRi approach, deletion of the distal upstream region by CRISPR/Cas9 most significantly repressed the expression of both *SFTPA1* and *SFTPA2* in A549 cells (Supplementary Fig. 8).

### Four regions at the *MYBPH* locus bound by NKX2-1 are all critical for the expression of *MYBPH* induced by NKX2-1

**Fig. 6:**
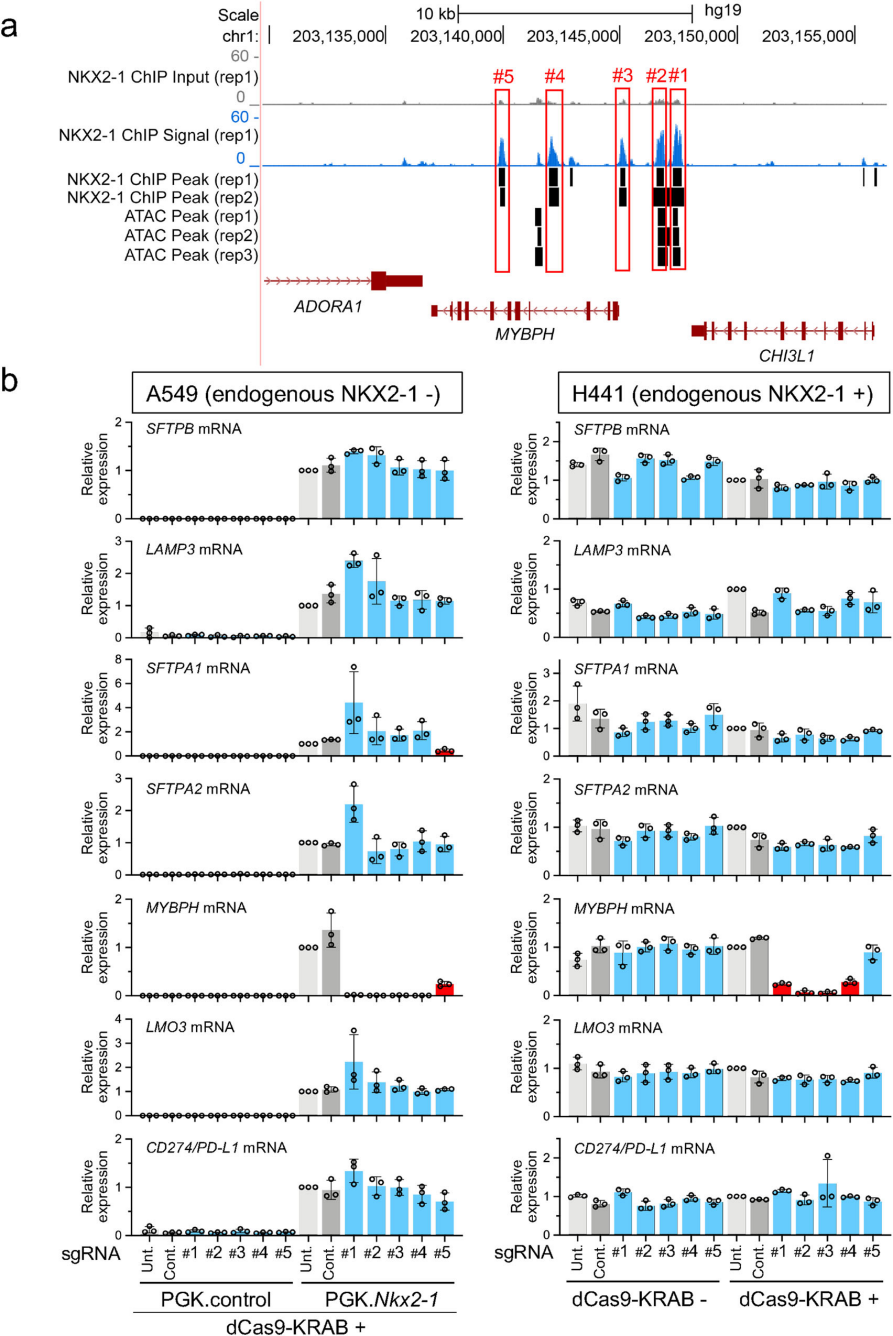
NKX2-1-binding sites located at three upstream and one intronic region are commonly required for the expression of *MYBPH*. **a** Shown are ATAC-seq and ChIP-seq data indicating that NKX2-1 binds to far distal (#1), distal (#2) and proximal (#3) upstream regions and the third (#4), and sixth (#5) intronic regions of the human *MYBPH* locus. Significant peaks are described as in Fig. 2a. **b** Shown is the gene expression of NKX2-1 downstream target genes affected by CRISPRi as described in Fig. 2b. The sgRNA targeting NKX2-1-binding sites located at three upstream (#1, #2, and #3) and one intronic (#4) regions significantly suppressed the expression of *MYBPH*. The sgRNA targeting NKX2-1-binding sites located at the sixth intronic (#5) region also significantly suppressed the expression of *SFTPA1* induced by NKX2-1 in A549 cells. Results are expressed as the mean ± SD of the triplicates for each group. Only *P* < 0.05 and more than twofold suppression are considered significant CRISPRi-mediated suppression (highlighted in red). Unt. untransfected, Cont. non-targeted control sgRNA.

The ChIP-seq data^27,30^ indicate that NKX2-1 binds to five regions (far distal, distal and proximal upstream, and third and sixth introns) of the locus of *MYBPH*, a product of which is involved in cell motility and metastasis of lung adenocarcinoma (Fig. 6a). Notably, two NKX2-1-binding sites located at the distal upstream regions (but not the three other regions) are in ATAC-accessible regions (Fig. 6a and Supplementary Data 1). As described above, the CRISPRi approach was conducted to assess the importance of these five regions (Supplementary Fig. 3). Notably, all sgRNAs targeting the five regions significantly suppressed the expression of *MYBPH* more than twofold (*P* < 0.05) in A549 cells (Fig. 6b), indicating that all five regions bound by NKX2-1 are essential for the expression of *MYBPH* that is induced by NKX2-1. The suppression of MYBPH was also confirmed at the protein level (Supplementary Fig. 4). Of note, the sgRNA targeting a region in the sixth intron significantly suppressed the expression of *SFTPA1* more than twofold (*P* < 0.05) (Fig. 6b) though the sgRNA sequence does not match the genomic DNA sequence at the *SFTPA1* locus according to the CRISPOR off-target search^38^ (Supplementary Data 6), suggesting that the sixth intron may regulate the expression of *SFTPA1* in *trans*. However, the sixth intron did not regulate the expression of either *MYBPH* or *SFTPA1* in H441 cells (Fig. 6b), suggesting a cell type-specific gene regulation by NKX2-1.

### Three regions at the second intron of the *LMO3* locus bound by NKX2-1 are all critical for the expression of *LMO3* induced by NKX2-1

**Fig. 7:**
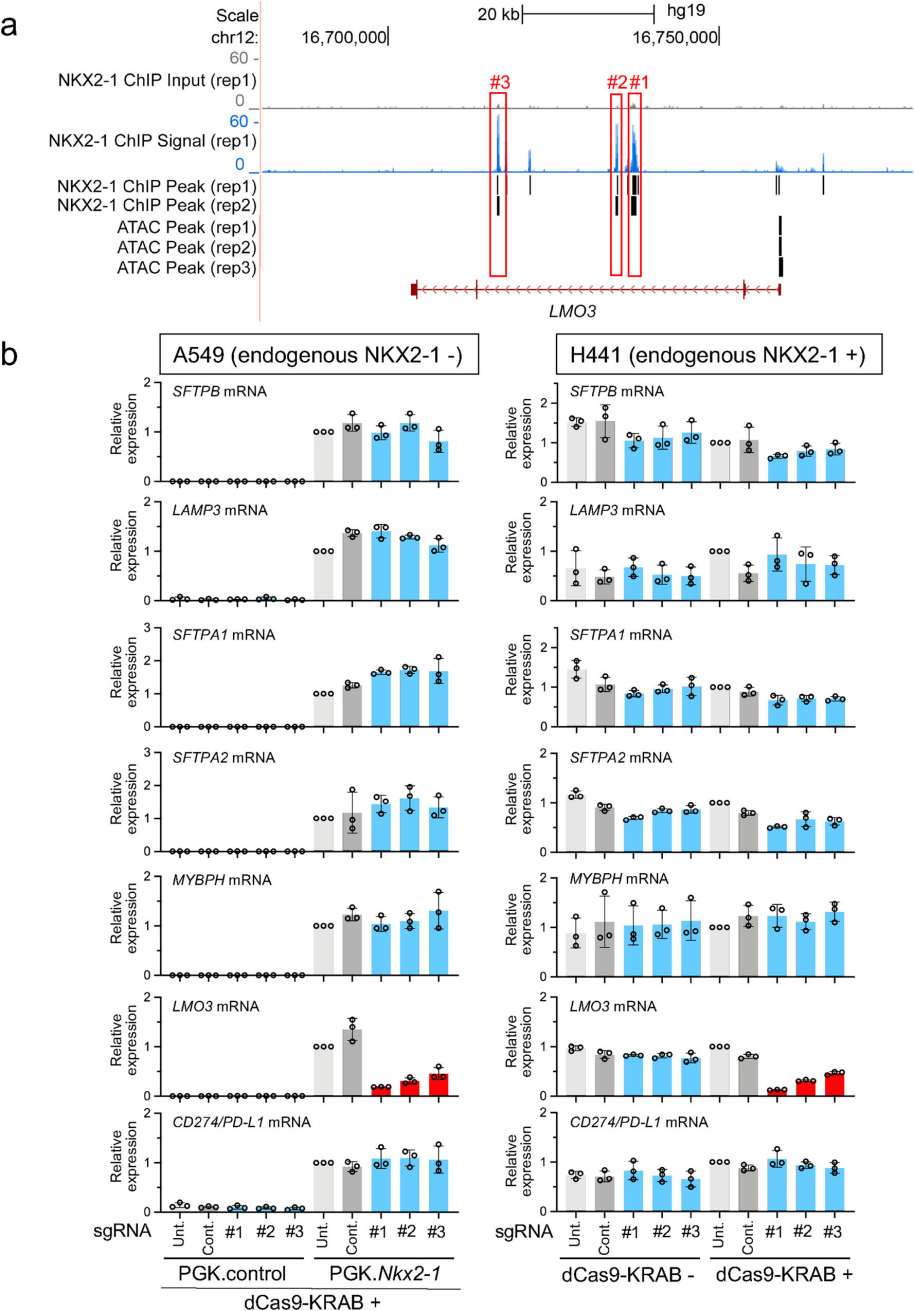
NKX2-1-binding sites located at three different regions of the second intron of *LMO3* are commonly required for the expression of *LMO3*. **a** Shown are ATAC-seq and ChIP-seq data indicating that NKX2-1 binds to three distinctive (#1, #2, and #3) regions of the second intron of human *LMO3*. Significant peaks are described as in Fig. 2a. **b** Shown is gene expression of NKX2-1 downstream target genes affected by CRISPRi as described in Fig. 2b. The sgRNAs targeting an NKX2-1-binding site located at the three distinctive (#1, #2, and #3) regions of the second intron of human *LMO3* significantly suppressed the expression of *LMO3*, respectively. Results are expressed as the mean ± SD of the triplicates for each group. Only *P* < 0.05 and more than twofold suppression are considered significant CRISPRi-mediated suppression (highlighted in red). Unt. untransfected, Cont. non-targeted control sgRNA.

The ChIP-seq data^27,30^ indicate that NKX2-1 binds to three distinct regions (ATAC inaccessible) at the second intron of *LMO3*, a product of which is involved in the survival of lung adenocarcinoma cells (Fig. 7a). As described above, CRISPRi was performed to assess whether these three distinct intronic regions influence the expression of *LMO3* (Supplementary Fig. 3 and Supplementary Data 7). Notably, all sgRNAs targeting the three regions significantly suppressed the expression of *LMO3* more than 2-fold (*P* < 0.05) in both A549 and H441 cells (Fig. 7b), indicating that all three regions at the second intron bound by NKX2-1 function as gene-regulatory regions for the expression of *LMO3* that is induced by NKX2-1, regardless of cell type.

### An upstream region but not the first intronic region bound by NKX2-1 is critical for the expression of *CD274/PD-L1* induced by NKX2-1

**Fig. 8:**
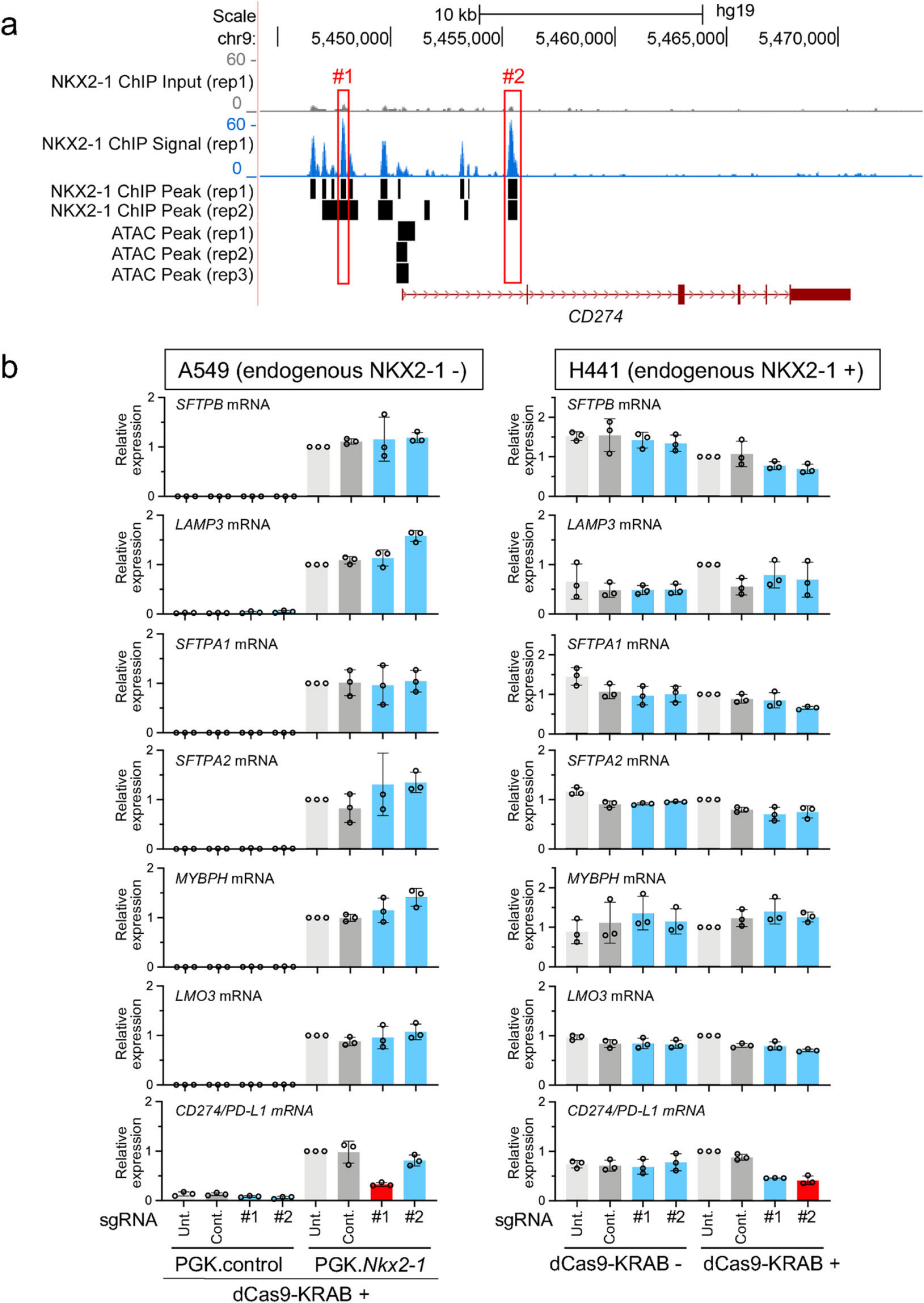
NKX2-1-binding sites located at the locus of *CD274/PD-L1* are required for the expression of *CD274/PD-L1* in a cell-type-dependent fashion. **a** Shown are ATAC-seq and ChIP-seq data indicating that NKX2-1 binds to the upstream (#1) and first intronic (#2) regions of human *CD274/PD-L1*. Significant ChIP-seq peaks are described as in Fig. 2a. **b** Shown is gene expression of NKX2-1 downstream target genes affected by CRISPRi as described in Fig. 2b. The sgRNA targeting an NKX2-1-binding site located at the upstream (#1) region significantly suppressed the expression of *CD274/PD-L1* induced by NKX2-1 in A549 cells while the sgRNA targeting an NKX2-1-binding site located at the first intronic (#2) region significantly suppressed the expression of *CD274/PD-L1* in H441 cells. Results are expressed as the mean ± SD of the triplicates for each group. Only *P* < 0.05 and more than twofold suppression are considered significant CRISPRi-mediated suppression (highlighted in red). Unt. untransfected, Cont. non-targeted control sgRNA.

The ChIP-seq data^27,30^ indicate that NKX2-1 binds to two regions (upstream and the first intron; ATAC inaccessible) of the locus of *CD274/PD-L1*, a product of which inactivates the anti-tumorigenic function of T cells (Fig. 8a). As described above, the CRISPRi approach was used to assess the gene-regulatory function of these two regions (Supplementary Figs. 3, 4 and Supplementary Data 8). Notably, an sgRNA targeting the upstream region significantly suppressed the expression of *CD274/PD-L1* more than twofold at the mRNA level (*P* < 0.05) and also at the protein level in A549 cells. In contrast, the sgRNA targeting the first intron did not suppress the mRNA expression of *CD274/PD-L1* (Fig. 8b), indicating that the upstream region bound by NKX2-1 functions as a gene-regulatory region for the expression of *CD274/PD-L1* and is indispensable while the intronic region in A549 cells is not. Although the siRNA-mediated loss-of-function study indicates that NKX2-1 does not regulate the expression of *CD274/PD-L1* in H441 cells, the sgRNA targeting the first intron of the *CD274/PD-L1* locus (an NKX2-1-binding site) significantly suppressed the expression of C*D274/PD-L1* more than twofold (*P* < 0.05) in H441 cells (Fig. 8b), suggesting that the first intronic region of *CD274/PD-L1* acts a critical gene-regulatory region in a cell-type-dependent fashion.

## Discussion

In the past decade, ChIP-seq has identified far more transcription factor-binding sites than gel-shift and reporter assays, and further has revealed that transcription factors bind not only proximal promoter regions but also non-proximal regions, including intronic regions and distal intergenic regions. However, none of these techniques can determine the functional roles of such regions. It is essential to determine whether such regions indeed influence the mRNA expression of genes located nearby and/or distally. The recent discovery of the CRISPR/Cas9 technology has resolved this issue by providing the means to delete or repress specific regions in order to determine their functional roles. Here, using the CRISPR/Cas9 technology, we determined the function of critical gene-regulatory regions bound by NKX2-1, a lineage-specific transcription factor that is essential for lung function and that has been most extensively studied in the lung field.

*SFTPB* is the first lung epithelial gene that was reported as an NKX2-1 downstream target gene. Using gel-shift and reporter assays, NKX2-1 was shown to bind and activate a proximal promoter region located at the first intron of *SFTPB*^4,5^, the data of which are consistent with our ChIP-seq and CRISPRi results (Fig. 2). Of note, this first intronic region contains a SNP (rs3024791) that is associated with chronic obstructive pulmonary disease (COPD)^39^, suggesting that this region is a critical region regulated by NKX2-1 that influences disease severity by affecting the expression of *SFTPB*. In addition to this region located at the first intron, using the ChIP-seq with CRISPRi approach, we identified another functionally important NKX2-1-binding site in the upstream region (Fig. 2). Likewise, SNPs located in this region may affect NKX2-1-binding and in turn influence the expression of *SFTPB*, thereby modifying the lung disease severity. Using the ChIP-seq assay, we previously identified three additional NKX2-1-binding sites in the ninth intronic, proximal, and distal downstream regions; however, the CRISPRi analysis in the present study indicated that two regions were cell-type-specific regulatory regions and one region was functionally redundant for the expression of *SFTPB* (Fig. 2), suggesting that all of the NKX2-1-binding sites identified by ChIP-seq are not always functionally critical in every cell type.

*LAMP3* is one of the lung epithelial genes highly induced by NKX2-1^6,27^. However, the molecular mechanism by which NKX2-1 regulates the expression of *LAMP3* has not been previously determined. Our ChIP-seq data indicate that NKX2-1 does not bind to a conventional binding region such as upstream or the first intronic region; instead, it binds to the third and fifth intronic regions that are more than ~10 kb away from the transcription start site (TSS). Furthermore, the CRISPRi data indicate that both regions are critically important for the expression of *LAMP3* induced by NKX2-1 (Fig. 4), indicating that ChIP-seq with CRISPRi is superior in identifying the gene-regulatory functions of these far distal regions compared to the conventional approach using gel-shift and reporter assays since they are normally limited to targeting regions within 5 kb from the TSS. Importantly, such a gene-regulatory mechanism was found only in one lung epithelial cell line (A549 cells) but not in the other lung epithelial cell line (H441 cells), indicating a cell-type-specific lung gene regulation.

The transcriptional regulation of *SFTPA/Sftpa* by NKX2-1 was initially studied using gel-shift and reporter assays to analyze mouse and baboon proximal promoter regions^40,41^. Later, human *SFTPA1* and *SFTPA2* gene regulation by NKX2-1 was reported by our group and others, focusing on their proximal promoter regions, using gel-shift and reporter assays^7–9^. Our ChIP-seq data indicate that NKX2-1 bound to the same proximal promoter region located at the upstream region of *SFTPA1* (Fig. 5a) and the CRISPRi data indicate that the proximal promoter region is indispensable for the expression of *SFTPA1* (Fig. 5b), which is consistent with our previous report using gel-shift and reporter assays^8^ and also suggests that this proximal region is conserved as an important gene-regulatory region among species. Unexpectedly, our ChIP-seq data also indicate that NKX2-1 binds to a distal upstream region of *SFTPA1* and *SFTPA2* (intergenic region between *SFTPA1* and *SFTPA2*), which is ~20 kb away from both genes (Fig. 5a). Importantly, this distal region was critical for the expression of both *SFTPA1* and *SFTPA2*, while the proximal region close to *SFTPA1* was critical only for the expression of *SFTPA1* but not *SFTPA2* (Fig. 5b), which is consistent with our previous finding^36^ that some gene-regulatory regions function to control the expression of a single gene while the others function to control that of multiple genes. Notably, these gene-regulatory mechanisms of *SFTPA1* and *SFTPA2* by NKX2-1 were seen in both A549 and H441 lung epithelial cell lines, suggesting that some lung gene-regulatory regions are highly conserved regardless of cell type.

Since the functional relevance of NKX2-1 in lung tumorigenesis other than as a biomarker was reported^19–22^, downstream target genes regulated by NKX2-1 that influence lung tumorigenesis have been actively sought (e.g., MYBPH, LMO3, and CD274/PD-L1). MYBPH is induced by NKX2-1 and acts as a tumor suppressor^24^. The molecular mechanism by which NKX2-1 regulates the expression of *MYBPH* was previously studied using reporter assay and ChIP-qPCR focusing on its promoter region located at the proximal upstream region, which indicates that NKX2-1 activates the promoter region^24^. This result is consistent with our ChIP-seq and CRISRPi data targeting the same region (Fig. 6). Importantly, our ChIP-seq and CRISRPi data indicate that not only the proximal upstream region but also three other regions located at distal upstream and intronic regions that are bound by NKX2-1 are functionally critical for the expression of *MYBPH* (Fig. 6). The sixth intronic region that is bound by NKX2-1 was critical in A549 cells but not in H441 cells. These results suggest again that an unbiased approach using ChIP-seq and CRISRPi in multiple cell types, which can search beyond the proximal regions near the TSS, helps to better understand the entire molecular mechanism by which transcription factors, including NKX2-1, regulate the expression of genes.

In contrast to MYBPH, which acts as a tumor suppressor, LMO3 (an NKX2-1 downstream target gene) promotes the survival of lung tumor cells^28^, indicating a context-dependent function of NKX2-1 in lung tumorigenesis. The molecular mechanism by which NKX2-1 regulates the expression of *LMO3* was previously studied using gel-shift and ChIP-seq assays, which indicated that NKX2-1 bound to a region located at the first intron of *LMO3*^28^, which is not consistent with our ChIP-seq data, indicating that NKX2-1 binds to three regions at the second intron of *LMO3* (Fig. 7a). This discrepancy might be due to the cell line that we used for ChIP-seq, which carries different oncogenes (H3122 cells carrying an *ALK* fusion [28] vs. A549 cells carrying *KRAS*^*G12S*^ [27]). Our CRISPRi data indicate that all three regions located at the second intron are essential for the expression of *LMO3* in both A549 and H441 cells (Fig. 7b; of note, H441 cells also carry a *KRAS* mutation, *KRAS*^*G12V*^, but not an *ALK* fusion), suggesting that the same CRISPRi approach targeting the region located at the first intron in H3122 cells would address the functional relevance of the region.

CD274/PD-L1 that is induced by NKX2-1 has been a hallmark of cancer immunotherapy along with its receptor PD-1 (also known as PDCD1), which can be targeted by therapeutic antibodies that activate anti-tumorigenic T cells by blocking the interaction between CD274/PD-L1 and PDCD1/PD-1^31^. The gene-regulatory mechanism of *CD274/PD-L1* has been described previously in reviews^42,43^. Our ChIP-seq data indicate that NKX2-1 binds to two regions of the *CD274/PD-L1* locus, ~ 2.7 kb upstream region and +4.8 kb first intron (Fig. 8a). To our knowledge, the upstream region is bound by only NKX2-1 while the intronic region is bound by not only NKX2-1 but also the AP-1 transcription factor^44^. Our CRISPRi data indicate that the upstream region is critical for the expression of *CD274/PD-L1* induced by NKX2-1 in A549 cells while the intronic region is critical for the expression of *CD274/PD-L1* in H441 cells (Fig. 8b). Since the siRNA-mediated loss-of-function analysis indicates that NKX2-1 is redundant for the expression of *CD274/PD-L1*, the intronic region bound by AP-1 might be critical for the expression of *CD274/PD-L1* in H441 cells.

In the present study, taking advantage of ENCODE database that harbors ATAC-seq datasets from A549 cells, we assessed whether NKX2-1 binds to chromatin-accessible genomic regions that are defined as such by ATAC-seq. To our surprise, more than half of the NKX2-1-binding sites were located at genomic regions that are considered to be chromatin inaccessible by ATAC-seq (Fig. 3a). To our knowledge, NKX2-1 is not considered to be a pioneer transcription factor that is able to bind to chromatin-inaccessible genomic regions^45^. Future studies are required to understand the mechanism by which NKX2-1 accesses such chromatin-inaccessible genomic regions or to develop a means to assess chromatin accessibility other than ATAC-seq.

Here, we investigated the roles of NKX2-1-binding sites in the expression of NKX2-1-regulated genes in human lung epithelial cells in vitro. Among such NKX2-1-regulated genes, the in vivo roles of *SFTPB* and *SFTPA1/SFTPA2* in lung pathophysiology have been particularly well studied using *Sftpb*- or *Sftpa1*-knockout mice^3,10–15^. Notably, a region bound by NKX2-1 in the first intron of human *SFTPB* is highly conserved with the one in the upstream region of mouse *Sftpb* (84% similarity). Likewise, a region bound by NKX2-1 in the proximal upstream region of *SFTPA1* is highly conserved with the one in the upstream region of mouse *Sftpa1* (74% similarity). Using CRISPR/Cas9-mediated deletion, Simeonov et al. have assessed the in vivo role of a region in the first intron of mouse *Il2ra* in blood cells, a region of which is highly conserved with a gene-regulatory enhancer region of human *IL2RA*^46^. In future studies, targeting such conserved gene-regulatory regions in mice using CRISPR/Cas9 and/or dCas9 will further determine the importance of NKX2-1-binding sites as gene-regulatory regions in lung pathophysiology in vivo.

## Methods

### Processing and visualization of ChIP-seq and ATAC-seq data

ChIP-seq of NKX2-1 in an A549 human lung epithelial (carcinoma) cell line, which we previously performed twice independently^27,30^ (GSE86959), was visualized using the UCSC Genome Browser^47^. Supplementary Figs. 9–14 describe both independent replicates with different gene isoforms. Peak files of three replicates of ENCODE ATAC-seq on A549 cell line (ENCODE accession#: ENCSR220ASC) were downloaded from GSE114202. ATAC peak genome coordinates were converted from GRCh38 to hg19 using the UCSC liftOver tool and visualized using the UCSC Genome Browser. Peak overlap was performed using HOMER mergePeaks function with “-d given” parameter^48^. Overlapped peaks among the three ATAC-seq replicates were considered as ATAC-seq peaks, and overlapped peaks between the two NKX2-1 ChIP-seq replicates were considered as NKX2-1 ChIP-seq peaks.

### Processing and visualization of Hi-C data

Mapping quality thresholded chromatin interactions (.hic files) of four replicates of ENCODE Hi-C on an A549 cell line (ENCODE accession#: ENCSR662QKG) were downloaded from ENCODE web portal. Inter-chromosomal interactions with the *SFTPB* locus (including 10 kb upstream of the transcription start site and 10 kb downstream of the transcription end site) were extracted at the resolution of 50 kb using the “dump” function of Juicer tools (version 1.22)^49^. Inter-chromosomal interactions with at least two observed counts were visualized in a circular layout using BioCircos^50^.

### Synthetic sgRNA

Synthetic sgRNAs targeting each locus (*SFTPB, LAMP3, SFTPA1, SFTPA2, MYBPH, LMO3*, and *CD274/PD-L1*; Supplementary Fig. 3 and Supplementary Data 3-8) were designed using CRISPOR^37^ and generated using the Invitrogen custom TrueGuide gRNA (sgRNA) ordering tool (ThermoFisher, Waltham, MA). Non-targeted gRNA (sgRNA) was used as a negative control (cat# A35526, ThermoFisher).

### Cells

A549 lung carcinoma cells and H441 lung epithelial (papillary adenocarcinoma) cells (obtained from ATCC in Manassas, VA) that stably express dCas9-KRAB were created and maintained as ATCC instructed^36^. A549 cells stably expressing dCas9-KRAB were infected by lentivirus carrying either rat *Nkx2-1* or backbone control (PGK-IRES-EGFP)^51^. A549 and H441 cells expressing dCas9-KRAB were transfected with synthetic sgRNAs (50 nM at the final concentration) or siRNAs targeting human *NKX2-1* (Silencer Select siRNA, ID: s224731 for #1, ID: s14154 for #2) or human *SFTPB* (Silencer Select siRNA, ID: s12757 for #1, ID: s227103 for #2) (60 nM at the final concentration; cat# 4390843 for negative control [siNeg]) using Lipofectamine RNAiMAX Reagent (cat# 13778075, ThermoFisher).

### Gene expression analyses

Gene expression analyses were performed using TaqMan gene expression analysis^36^ using probes (Hs00167036 for *SFTPB*, Hs01111316 for *LAMP3*, Hs00831305 for *SFTPA1*, Hs00359837 for *SFTPA2*, Hs00192226 for *MYBPH*, Hs00998696 for *LMO3*, Hs01125301 for *CD274/PD-L1*, Rn01512482 for rat *Nkx2-1*, Hs00968940 for human *NKX2-1* and Hs02758991 for *GAPDH* as control from ThermoFisher). A probe for *dCas9-KRAB* was custom designed by ThermoFisher as described in ref. ^36^.

### RNA-seq analysis

RNA-seq^36^ was performed, in which RNA was obtained from A549 cells constitutively expressing dCas9-KRAB that were transiently transfected with synthetic sgRNA targeting the first intron of *SFTPB* (#2) or non-targeted control gRNA. After quality assessment and pre-processing of RNA-seq reads^36^ were performed, gene expression was measured using htseq-count. Differential expression analysis for each comparison was performed using Bioconductor DESeq2 package. Differential expression with at least log2 fold change ≧1 or ≦−1 and padj<0.05 was considered significant.

### CRISPR/Cas9-mediated deletion of an NKX2-1-binding site

An NKX2-1-binding site located at the intergenic region of *SFTPA1* and *SFTPA2* was deleted in A549 cells using CRISPR/Cas9^30^, in which sgRNA target sequences described in Supplementary Fig. 8b were used. The deletion was confirmed by PCR amplification (Supplementary Fig. 8c) and Sanger sequencing by the DNA Sequencing and Genotyping Core at CCHMC using primers described in Supplementary Fig. 8b.

### Immunoblotting (IB)

Immunoblot assays^52^ were performed using rabbit anti-NKX2-1 (TTF-1) antibody (1:5,000, cat# WRAB-1231; Seven Hills Bioreagents, Cincinnati, OH), rabbit anti-MYBPH (1:5,000, cat# HPA061383; MilliporeSigma, St. Louis, MO), rabbit anti-CD274 (PD-L1) antibody (1:5,000; clone E1L3N; cat# 13684, Cell Signaling Technology, Danvers, MA), rabbit anti-Cas9 (dCas9) antibody (1:5,000; EPR18991; cat# 189380, Abcam, Cambridge, UK), and rabbit anti-ACTA1 antibody (1:5,000; cat# A2066, MilliporeSigma).

### Statistics and reproducibility

Realtime qPCR results were normalized against non-targeted sgRNA samples in independent biological triplicates. Results are expressed as the mean ± SD of the triplicates for each group. Undetermined Ct values due to low or no expression were set as Ct=40. Statistical relevance was determined using a two-sided Student’s *t*-test on transfected vs. control sgRNA transfected cells' relative expression with a minimum *P* value < 0.05. GraphPad Prism 8 was used for graphing and statistical analysis. Raw numbers for all replicates that generated the results are shown in Supplementary Data 9.

### Reporting summary

Further information on research design is available in the Nature Research Reporting Summary linked to this article.

## Supplementary information

Supplementary information

Description of Additional Supplementary Files

Supplementary Data 1–9

Reporting Summary

## Data Availability

The authors declare that all data supporting the findings of this study are available within the article and supplementary data, including Supplementary Figs and Supplementary Data. The RNA-seq raw data are available in the GEO repository (Gene Expression Omnibus, https://www.ncbi.nlm.nih.gov/geo/, accession number: GSE162186).
