# Supplementary material for: CRISPRi-mediated functional analysis of NKX2-1-binding sites in the lung
Source: Commun Biol. 2021 May 12;4:568. doi: 10.1038/s42003-021-02083-4 (PMC8115294; doi:10.1038/s42003-021-02083-4)
Supplement: Supplementary file 1 — Supplementary information [file 42003_2021_2083_MOESM1_ESM.pdf]

Supplementary Information

CRISPRi-mediated functional analysis of NKX2-1-binding sites in the lung

William Stuart, Iris M. Fink-Baldauf, Koichi Tomoshige, Minzhe Guo and

Yutaka Maeda

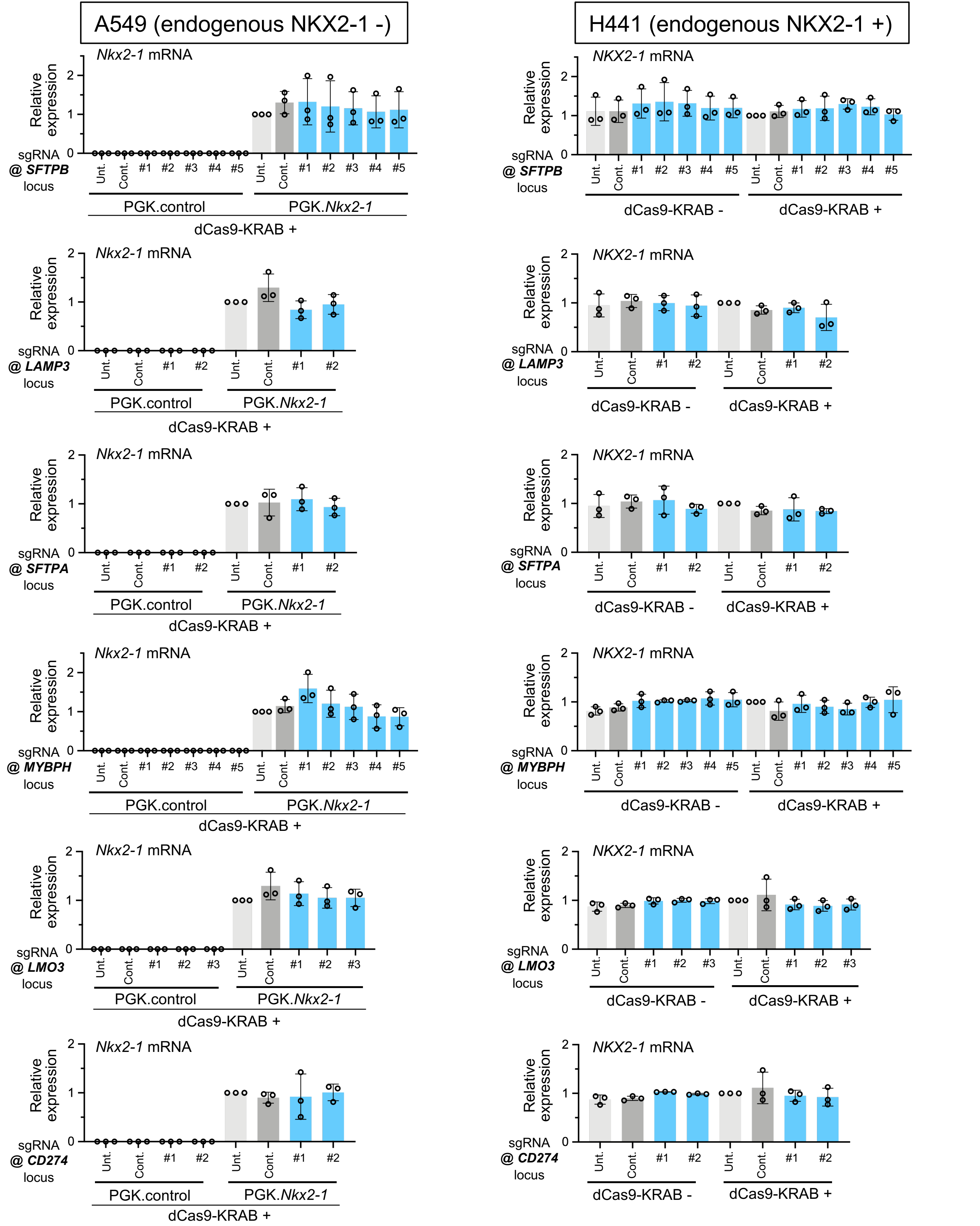

Supplementary Fig. 1. The expression of *Nkx2-1/NKX2-1* is not altered by synthetic sgRNAs in the presence of dCas9-KRAB in A549 and H441 lung epithelial cells

Shown is gene expression of rat *Nkx2-1* in A549 cells and human *NKX2-1* in H441 cells, both of which stably express dCas9-KRAB. A549 cells expressing dCas9-KRAB, which we previously created (1), were infected with lentivirus carrying rat *Nkx2-1* (PGK.*Nkx2-1*) or empty (PGK.control). Likewise, H441 cells expressing dCas9-KRAB were created. TaqMan gene expression analysis was performed using mRNA from these cells as described in Methods. The expression of *Nkx2-1*/*NKX2-1* was not influenced by indicated synthetic sgRNAs. Results are expressed as the mean ± SD of the triplicates for each group. Unt., untransfected. Cont., non-targeted control sgRNA.

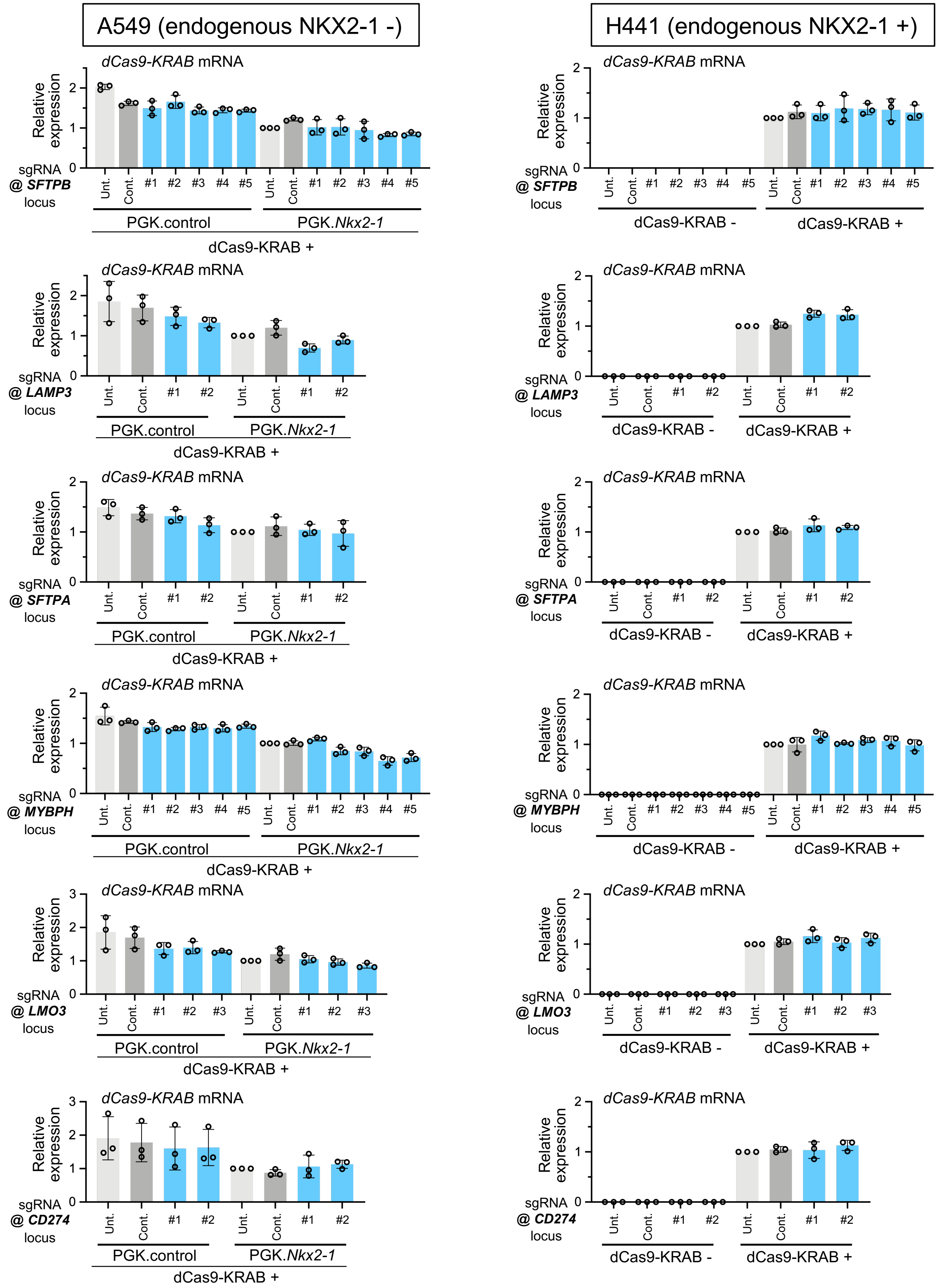

Supplementary Fig. 2. The expression of *dCas9-KRAB* is not altered by synthetic sgRNAs in A549 and H441 lung epithelial cells
Shown is gene expression of *dCas9-KRAB* in A549 cells stably expressing *dCas9-KRAB* with or without *Nkx2-1* and H441 cells stably expressing *dCas9-KRAB*. TaqMan gene expression analysis was performed using mRNA from these cells as described in Methods. *dCas9-KRAB* expression was not influenced by indicated synthetic sgRNAs. Results are expressed as the mean ± SD of the triplicates for each group. Unt., untransfected. Cont., non-targeted control sgRNA.

Supplementary Fig. 3. sgRNA target sequences for regions that harbor NKX2-1-binding sites

NKX2-1-binding motif: **cttg** or **caag**
sgRNA target sequence: xxxxxxxxxxxxxxxxxxxx
PAM sequence: xxx
SNP: x

SFTPB locus

Upstream region #1

cctcatcatgcccctcaggactcca**cttg**aggcctcttacttcagggagagac**caag**aag

First intronic region #2

SNP rs3024791 (C/T or G/A): g

gtaaggaggtggtgagagtgtcctgggtctgcccttccaggg**cttg**ccctgggttaagagccaggcaggaagctct**c**
aagagcattgct**caa**gagtagagggggcctgggaggcccagggaggggatgggaggggaacacccaggctgcccca
accagatgccctccaccctcctcaacctccctcccacggcctggagaggtgggaccaggtatggagg**cttg**agagcc
cctggttggaggaagcca**caag**tccaggaacatgggagtctgggcagggggcaaaggaggcaggaacaggccatcag
ccaggacaggtggtaaggcaggcaggagtgttcctgctgggaaaggtgggat**caag**cacctggagggctcttcaga
gcaaagacaaacactgaggtcgctgccactcctac

Ninth intronic region #3

gctgagtgagcaccagg**caag**ccttcagaggcctggttatctaccaggagatgagtaatgctagggccagtt**caag**c
caggaaagggactagccttctctccagggtcctgatccctttactgcccccacactcct**caag**gtgtgactcactca
ggacaaacccattggcaaaaggagagggctgga**cttg**aaggtcctagggcc**cttg**ccaatactcagtcaatgacagg
aaattccctttttttttttttttttttttttttgagatggagttttgct**cttg**ttgcccaggctggagtgcaatgg
cacaat**cttg**gctcactgcaacctctgcctccgggttcaggcgattctcctgcctcagcct**cttg**agtagctgggat
tacaggcatgtgctaccaggcccggctaattttg

Proximal downstream region #4

gtaagagcccacggttggggatgaatctgtgacagatttagtggat**cttg**agatgagcagaaatcagatgtgtagca
gtgatggca**caag**aagatgatgatcagg**caag**acgagagacactccagagagacctggatggccagggaatgcttag
tccccagtgcctctcctcaccgcagggcaatctgtaaatcattcccagggaaaggggaaagaggattcagtggttag
tcagc**caag**

Distal downstream region #5

cctgtaatcccagtactttaggaggccgaggcgggcagatca**cttg**agctcagaagtttgaggccagcctgggcaac
atggtgaaacaccaccactacaaaaaaaatcaaaacaattagcccagtgtagtggtg**caag**cctgtgggcccagcta
ctcaggaggctgaggtgggaggatgg**cttg**agcccaggaggcggagattgcagtgagc**caag**atcatgccactacac
tccagcctg
ggcgacagtgccagaccctgtctcaaaaaaaaaaaaaaaaaaaaaaaaaaaaaagtagaaacttcctgataaattag
gctagcagctgaggatttca**cttg**ctatcctaagagccagtgaagtgtctttaagcatgagatcagagtatgggga
gaaatgactcaggtttgtgttttaaaagattactctgactaaagcatggataaactgggggtaggaggcaaagaaat
gtggagagacctattgagagcactgcaaacagaaactggcaatagcttaaactaaaatgatagtggcaaagggccag
tacagaagtctgatggat**cttg**gagaacgaccgtggactatcgtaaacttaa

LAMP3 locus

Third intronic region #1

ctccttctgtgcagcccaaaaagggcttcagggcctcttatgggaga**cttg**acctctggggaacgtaaccacccgtg
ccctttgcaaaccagccaaaggaggaagttgtgcagatgaata

Fifth intronic region #2

gacaatctgatatgaggacccgggagttccccaaagagactcgggcttaacagcaagag**cttg**gctttggagagctg
tgatctccttccacctcctccttcaaactggcacctgctccaggctcccctgtgtccagtgttaagggcactcgaaa
gttctgtagggcagagga**caag**gtgtttattcaccagccactcactcttctgcagaatgattaatgtaagatttacc
ttctagcatgtggaaatttctcccctagcagatgaaa**caag**aagcagaatttaaaat**caag**tatagagg

SFTPA (*SFTPA1* and *SFTPA2*) locus

Proximal upstream region #1

gtgcctgtgtaagggctctcggctttcactgctaaggag**cttg**ctaggggcagggtgggtgacctcagc**caag**gggg
ggatttctcttcttacagacctggagttcctctttcgcaggttctgtgctcccct**caag**ggtccttt gagatcctcc
agcctgagtgct**cttg**gggaaacatg

Distal upstream region #2

aggtggggagtggggtgggtcacttccctgtgactctgc**cttg**tgataggcattttggcttct**caag**ggtcctcacc
cttgtgtactcatgtcatcaggccctgtccagcggccctcccggttcccattcagggggcctgccaggcac**caag**ag
gtgcttccgtggtaaagaagatccctct**caag**gctgtgtctcctgatgccattgacacaatgttgaagagcccatag
gcccagagg

MYBPH locus

Far distal upstream region #1

cgggactggagtccctgcagagtg**cttg**agagatggccctccagcctcccagtgaggggcagccgattac**cttg**gaa
gcaacccattccgtggctgggctgaaatctgtcaatagtcctagtgctgggcttagccctttgttctgttcacccct
cccctaacactcatccctccacccattcagagatcaaagtacctctgct**caag**ctggcctgagataggaggtcggga
ggatgtgat

Distal upstream region #2

cccaccccaccacacaggcatctttgaaaccccagggctgcaagcc**cttg**tgggcagccaactgccctcccctgggg
ccacccttcccccagccggggacaggctctccagggctgactcagtgtcctgtggggttagtcacattcacttatag
ctgcagagtcagaggc**caag**gatggagatccagtctccaggacccactccctgcctgg**caag**acgtggagtcaccta
gcagagggc

Proximal upstream region #3

agatggatgaggaagtgactcct**caag**gggcc**cttg**tggttcctcccccagagctattcctggcctgggcgcctctc
caccctccagtcctcctg**cttg**acctgaccccacagctgggaa

Third intronic region #4

ggggccctgtccaggatc**cttg**gctgctcagctgtctctc**cttg**gccacctggagctgc**caag**catt**cttg**accccc
tgcagctgtccctagagcattccatgcctgagctcccagagc**caag**tgcccactcagctaatcctcagagacaccgt
gttccttctcactgt**cttg**tcctcct

Sixth intronic region #5

acctgactccctaggaggcccttag**caag**actcttcctctctggacctcagatgcctcctgaagactgtgaagaggg
ctggcccgggctgtctgaagccttt**caag**ttctggagccatgcttctcaaatcttaatgtccacatgagtcacctgg
ggag**cttg**ctaagaggcagagtctga

LMO3 locus

Second intronic region #1

ttgaaac**caag**ccctaa**cttg**ttgagcctc**cttg**accctactgaacatcagcctgccatc

Second intronic region #2

tttatagttttattctatgccaaaaatag**cttg**ttaggaatataaaagaaattaataaagagtagtaatacaattat
taaaatatatatgctttagagttcagcattcatatgaagaaatctttttaaaggaaaactctgctgaaaaaaaagtc
ctctatttttctgttcttccctgctagataagtatcacagaaaaatctcaatgagactgatggtaagcctgttataa
tagcaatggaattttgagtgtttgttttaacattgtgaaagaaagtcaacagaatgaaactcagatcatggcaaata
ttt**cttg**aggatttactctataatgggtagtgttataggaactgaggctccagcagttaaaaaaatggacagaaata
tttgcctttatgtatcttatattttagtattatttttcaggtattctcaatgagttatatatgtgctaagtgctgta
cagatgtaatctcctctagcc**cttg**ggaactacagtattattaaacccattttagagaagaaggcactgaggctgag
g

Second intronic region #3

a**caag**cactacaaa**caag**aattta**caag**gatctccagcttaaatggaaaagtggatctatgataactttataaacga
gtgtgggagtaaagagtgcagaggaagaggtta**caag**aattat

CD274/PD-L1 locus

Upstream region #1

aagat**caag**aacatttactggaaattgctccttcaccaggaatttgctcacatctcttcaggtccacttataagat**c**
ttgaaatcagtcctcctgagatcagtacaaacgaggatcatctctcctgctttagaagcaaaagtggcaagcagggt
agacagaggcagaaggaaggatggta

First intronic region #2

ggtgctcaatcagtgtttgctaaacgaaataattagtcacattt**caag**caggatgactaaatgaagaatagaatcta
ggcagatactctggaagagtggctgtgagtcattcatatctta

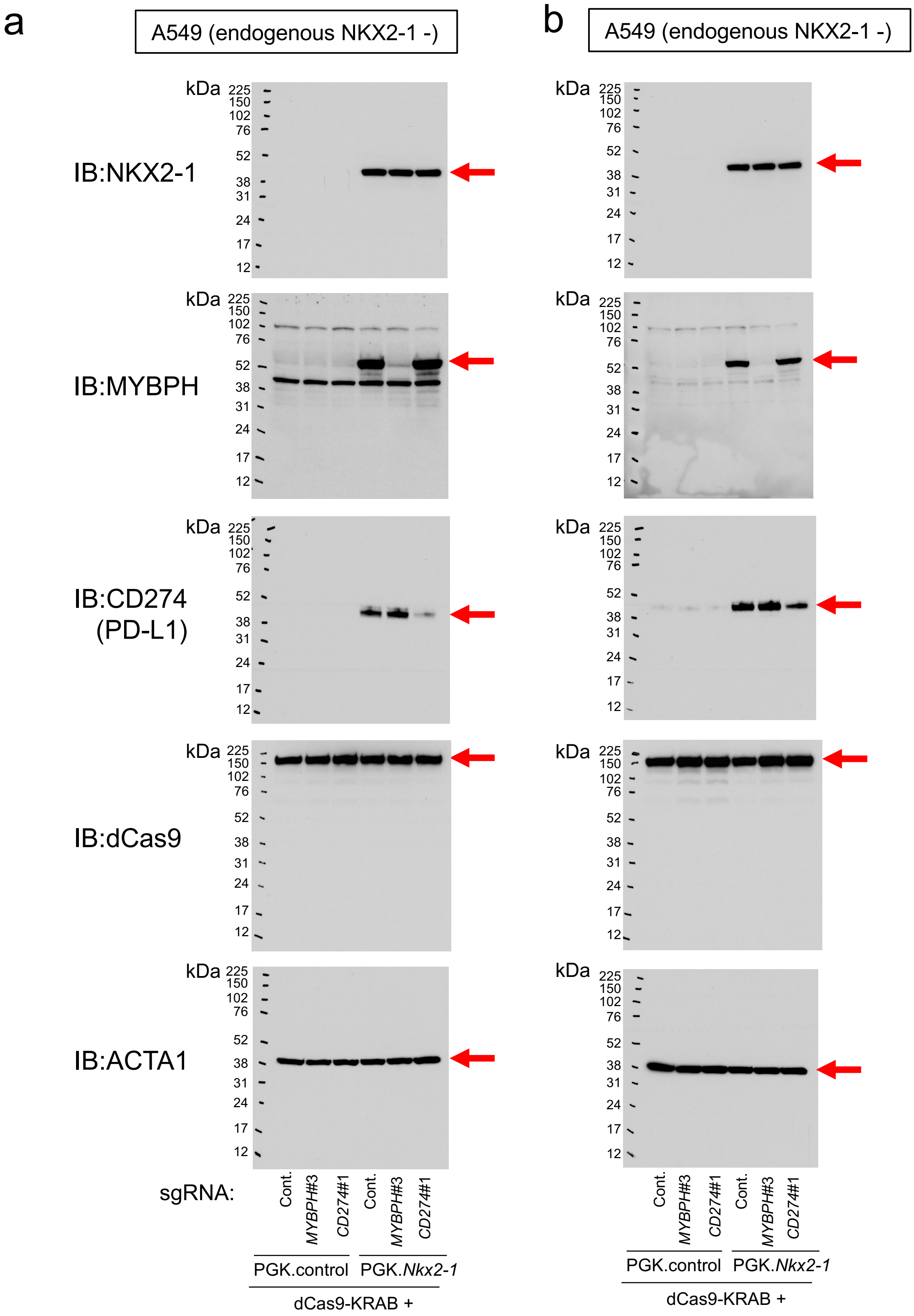

Supplementary Fig. 4. CRISPRi targeting NKX2-1-binding sites, including ones at loci of *MYBPH* and *CD274*, represses the expression of MYBPH and CD274 at the protein level in A549 lung epithelial cells that express NKX2-1

Shown are immunoblots of indicated proteins using protein extracts from A549 cells with or without NKX2-1 (PGK.rat *Nkx2-1* or PGK.control) that were performed independently twice (a and b). Red arrows indicate protein bands targeted by antibodies specific to the proteins. ACTA1 was used as a loading control. SgRNAs used in this analysis are indicated at the bottom. Cont., non-targeted control sgRNA.

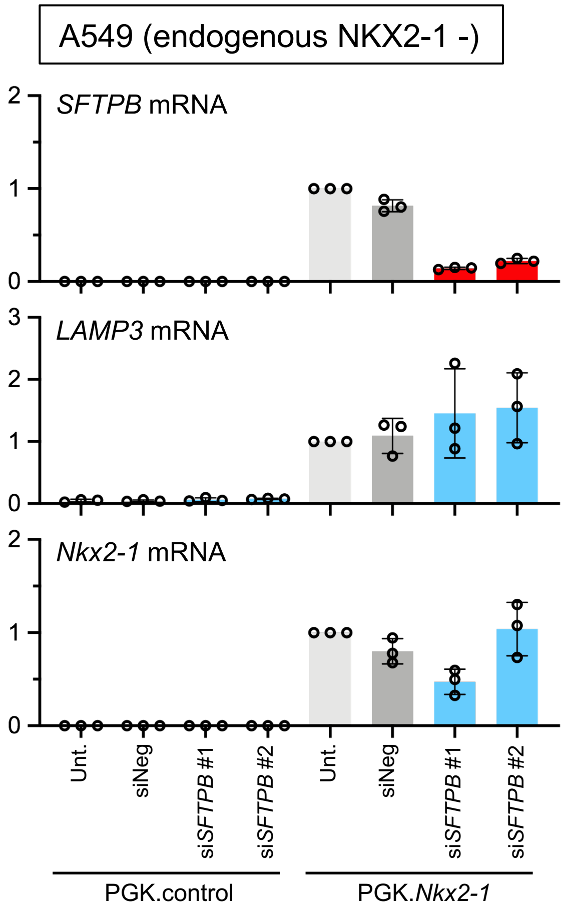

Supplementary Fig. 5. The expression of *SFTPB* does not affect the expression of *LAMP3* in A549 lung epithelial cells that express *Nkx2-1*

Shown is the expression of *Nkx2-1* and its downstream target genes, *SFTPB* and *LAMP3*, in A549 cells with or without *Nkx2-1* (lentiviral PGK.*Nkx2-1* or PGK.control, respectively), which were transiently transfected with two independent siRNAs targeting endogenous *SFTPB* (si*SFTPB* #1 or si*SFTPB* #2) or non-targeted negative control siRNA (siNeg). Unt. indicates untransfected. The data points are obtained from three independent experiments as described in Methods. Results are expressed as the mean ± SD of the triplicates for each group. Only P<0.05 and more than 2-fold suppression are considered significant siRNA-mediated suppression (highlighted in red).

a

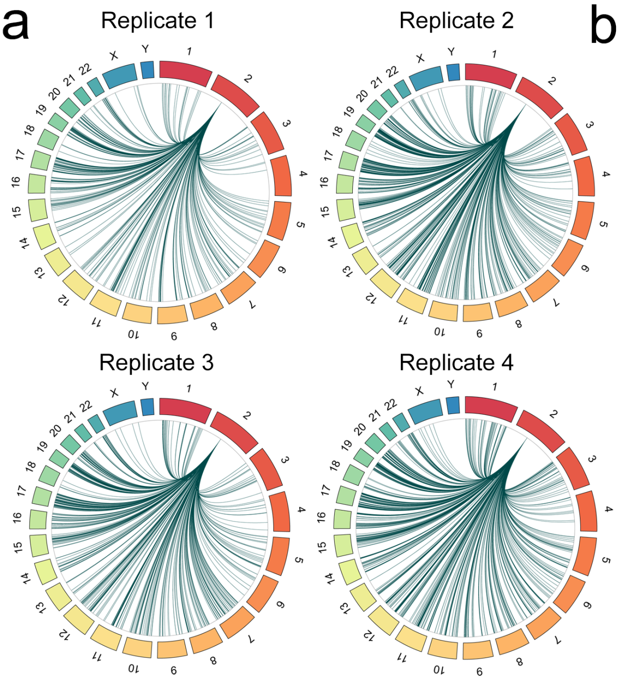

b

Number of Hi-C interactions with *SFTPB* locus in each chromosome

Chromosome	Replicate 1	Replicate 2	Replicate 3	Replicate 4
1	20	38	31	43
3	17	22	16	33
4	3	14	10	12
5	17	20	23	32
6	6	17	9	20
7	24	31	41	37
8	18	35	31	43
9	21	42	20	36
10	15	33	33	37
11	27	47	27	51
12	15	62	42	46
13	4	9	8	16
14	11	29	20	33
15	18	40	27	35
16	33	58	43	44
17	64	109	84	108
18	3	5	4	13
19	48	91	63	76
20	24	47	39	50
21	5	2	4	9
22	10	12	15	16
X	4	6	4	5
Y	0	0	0	0

Supplementary Fig. 6. The locus of human *SFTPB* on chromosome 2 interacts with genomic regions located on different chromosomes in A549 lung epithelial cells

a. Shown are Hi-C data of four isogenic replicates that indicate the interaction of the *SFTPB* locus with genomic regions located on different chromosomes. Hi-C data were obtained from ENCODE.

b. Shown are distributions of inter-chromosomal interactions in individual Hi-C data replicates. Inter-chromosomal interactions with the *SFTPB* locus (including 10 kb upstream of transcription start site and 10 kb downstream of transcription end site) were extracted at the resolution of 50 kb. Interactions with at least 2 observed counts were included in the analysis.

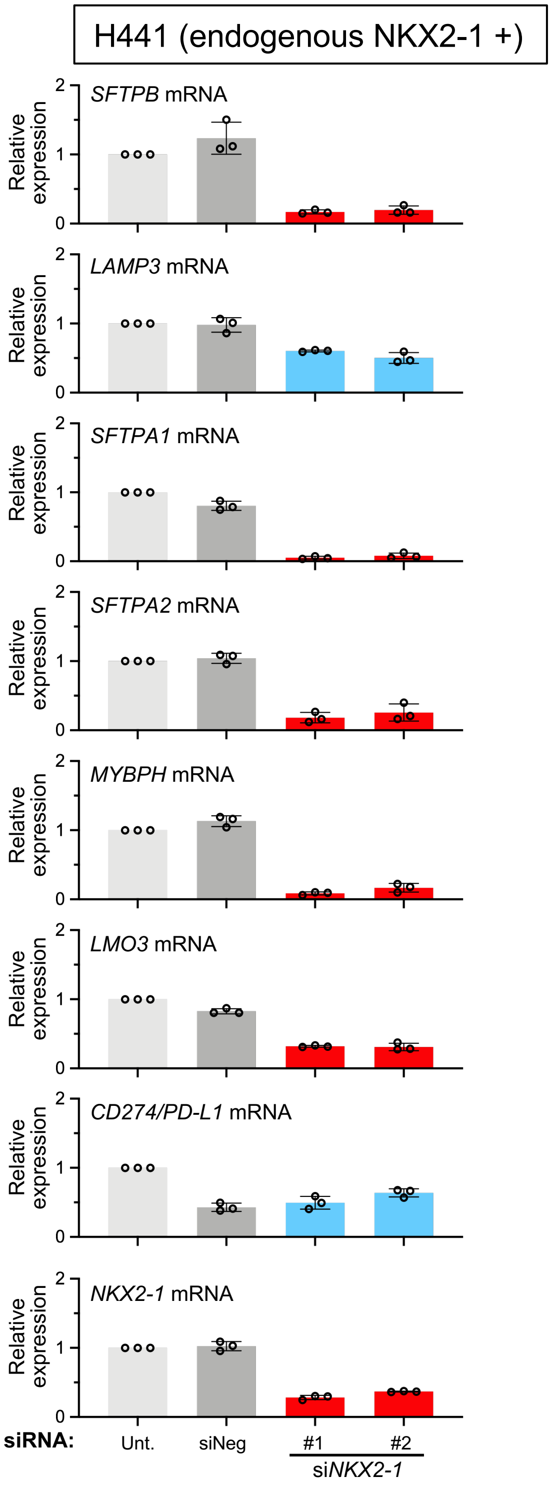

Supplementary Fig. 7. SiRNA-mediated suppression of *NKX2-1* indicates that NKX2-1 is required for the expression of *SFTPB*, *SFTPA1*, *SFTPA2*, *MYBPH* and *LMO3* in H441 lung epithelial cells

Shown is the expression of endogenous *NKX2-1* and genes in H441 cells, which are induced by rat *Nkx2-1* in A549 lung epithelial cells, transfected with two independent siRNAs (#1 and #2) targeting endogenous *NKX2-1*. siNeg is a negative control siRNA and Unt. indicates untransfected. The data points are obtained from three independent experiments as described in Methods. Of note, the expression of *LAMP3* and *CD274/PD-L1* is regulated by NKX2-1 in A549 cells but not in H441 cells. Results are expressed as the mean ± SD of the triplicates for each group. Only $P<0.05$ and more than 2-fold suppression are considered significant siRNA-mediated suppression (highlighted in red).

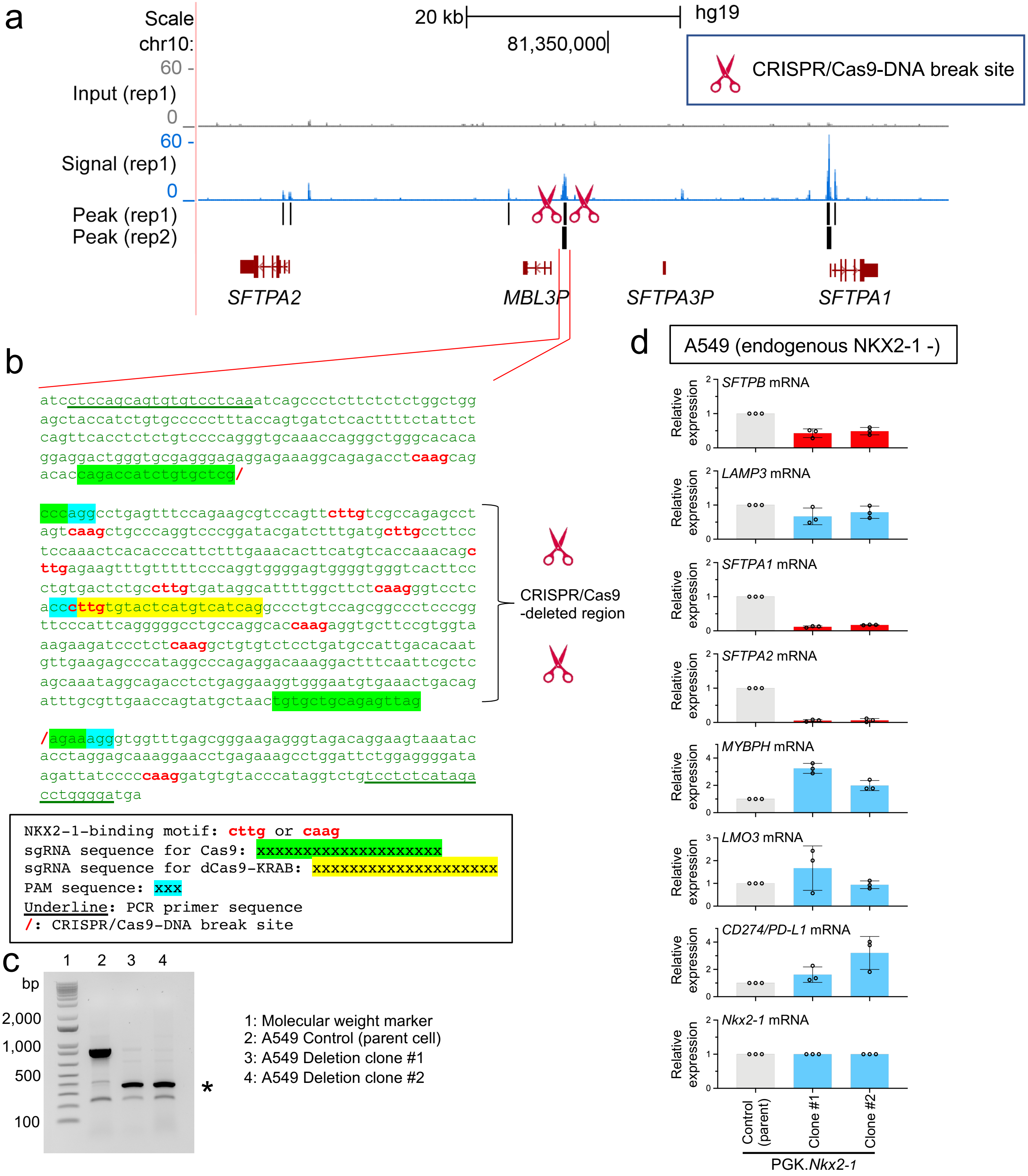

Supplementary Fig. 8. CRISPR/Cas9-mediated deletion of an NKX2-1-binding region located at an intergenic region between *SFTPA1* and *SFTPA2* represses the expression of *SFTPA1* and *SFTPA2* in A549 lung epithelial cells

a. Shown is ChIP-seq data indicating that NKX2-1 binds to an intergenic region of human *SFTPA1* and *SFTPA2*. Significant ChIP-seq peaks are described as in Fig. 5a. *MBL3P* and *SFTPA3P* are pseudogenes. The region of CRISPR/Cas9-mediated deletion is indicated by two scissors.

b. Shown is the sequence of genomic DNA that is deleted by CRISPR/Cas9. SgRNA sequence information is indicated.

c. Deletion was confirmed by PCR amplification using primers described in b. * indicates the deletion. Two independent clones were obtained as described in Methods.

d. Shown is gene expression analyzed as described in Fig. 2b using mRNA obtained from A549 cells (described in c) that were infected with lentivirus carrying rat *Nkx2-1*. Expression was normalized to the expression of *GAPDH* and *Nkx2-1*. Statistical significance was determined using Student's *t*-test with Welch's correction for unequal variances. Results are expressed as the mean ± SD of the triplicates for each group. Only $P<0.05$ and more than 2-fold suppression are considered significant (highlighted in red).

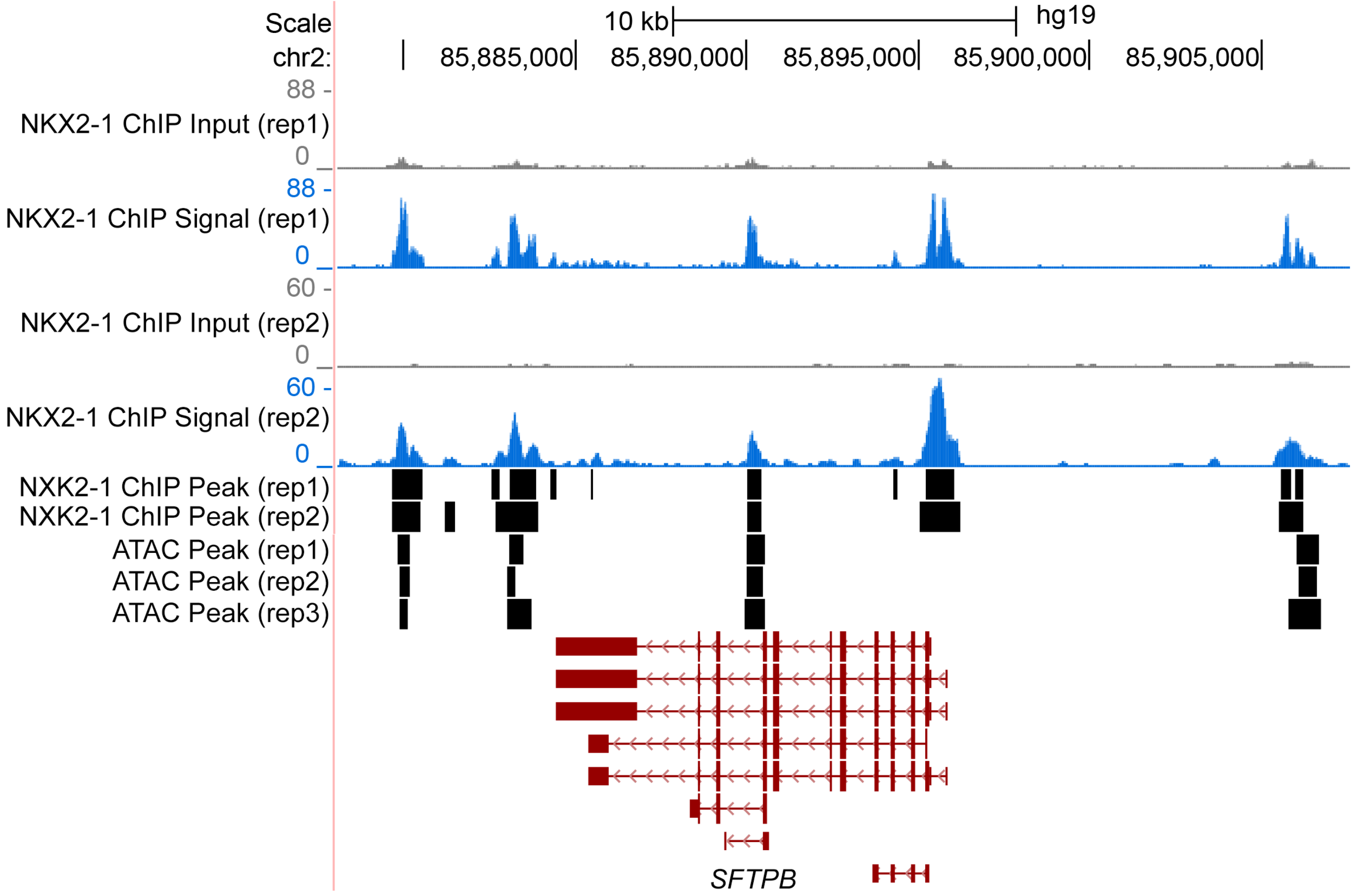

Supplementary Fig. 9. NKX2-1-binding sites at the human *SFTPB* locus were identified by two independent ChIP-seq analyses
Shown are ChIP-seq and ATAC-seq data from two or three independent experiments along with different isoforms of *SFTPB* as described in Fig. 2a.

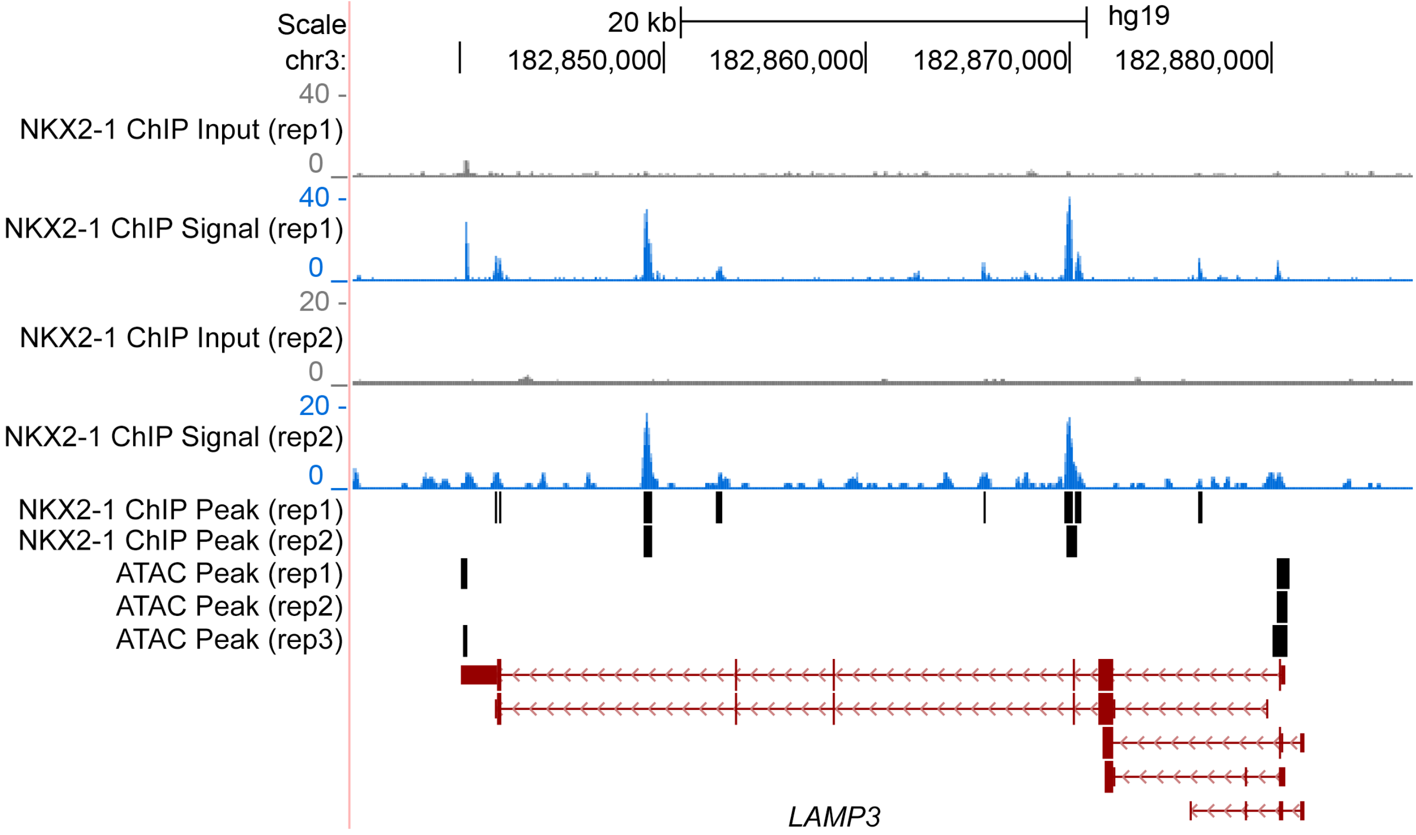

Supplementary Fig. 10. NKX2-1-binding sites at the human *LAMP3* locus were identified by two independent ChIP-seq analyses
Shown are ChIP-seq and ATAC-seq data from two or three independent experiments along with different isoforms of *LAMP3* as described in Fig. 4a.

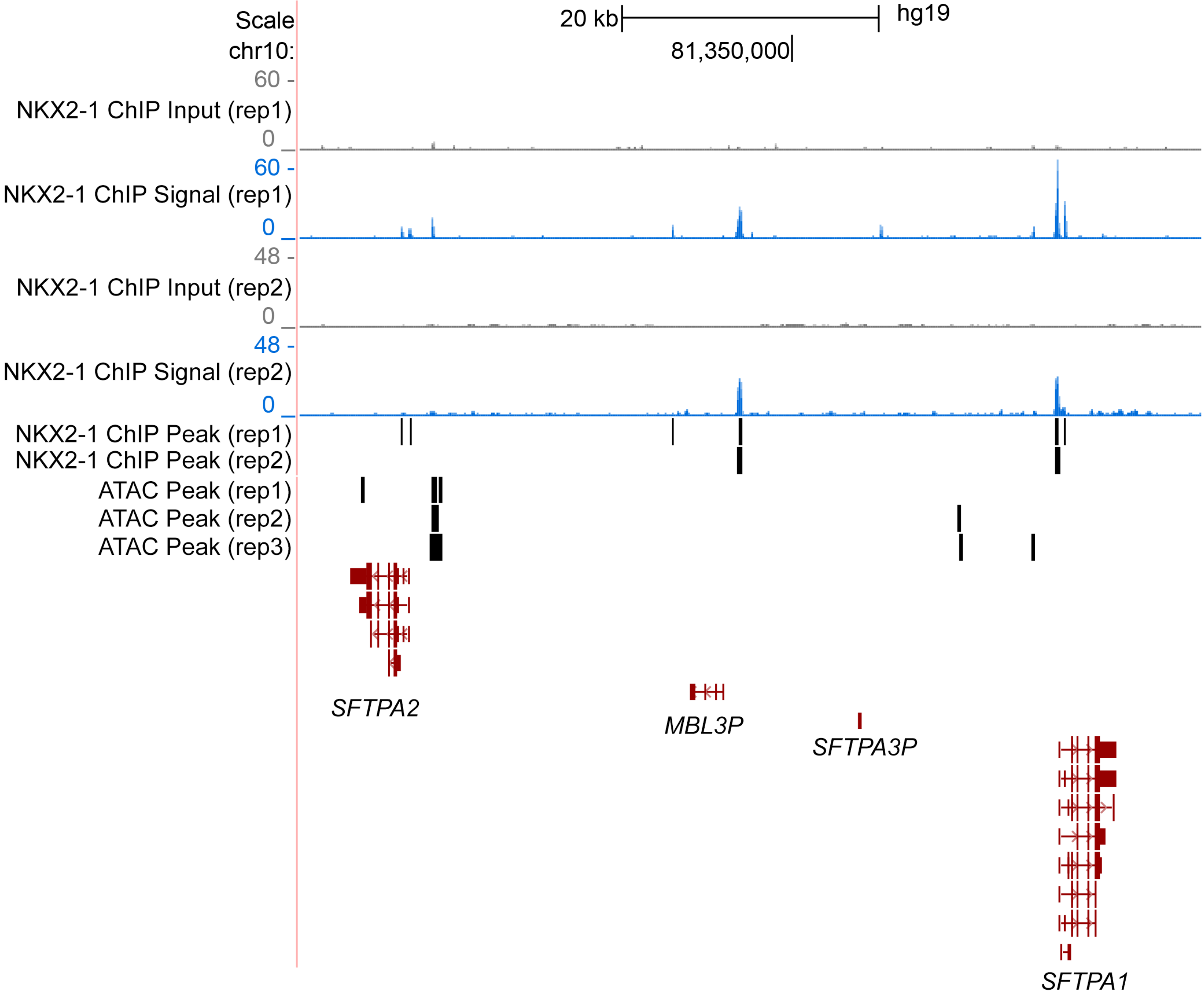

Supplementary Fig. 11. NKX2-1-binding sites at the human *SFTPA* locus were identified by two independent ChIP-seq analyses
Shown are ChIP-seq and ATAC-seq data from two or three independent experiments along with different isoforms of *SFTPA1* and *SFTPA2* as described in Fig. 5a. *MBL3P* and *SFTPA3P* are pseudogenes.

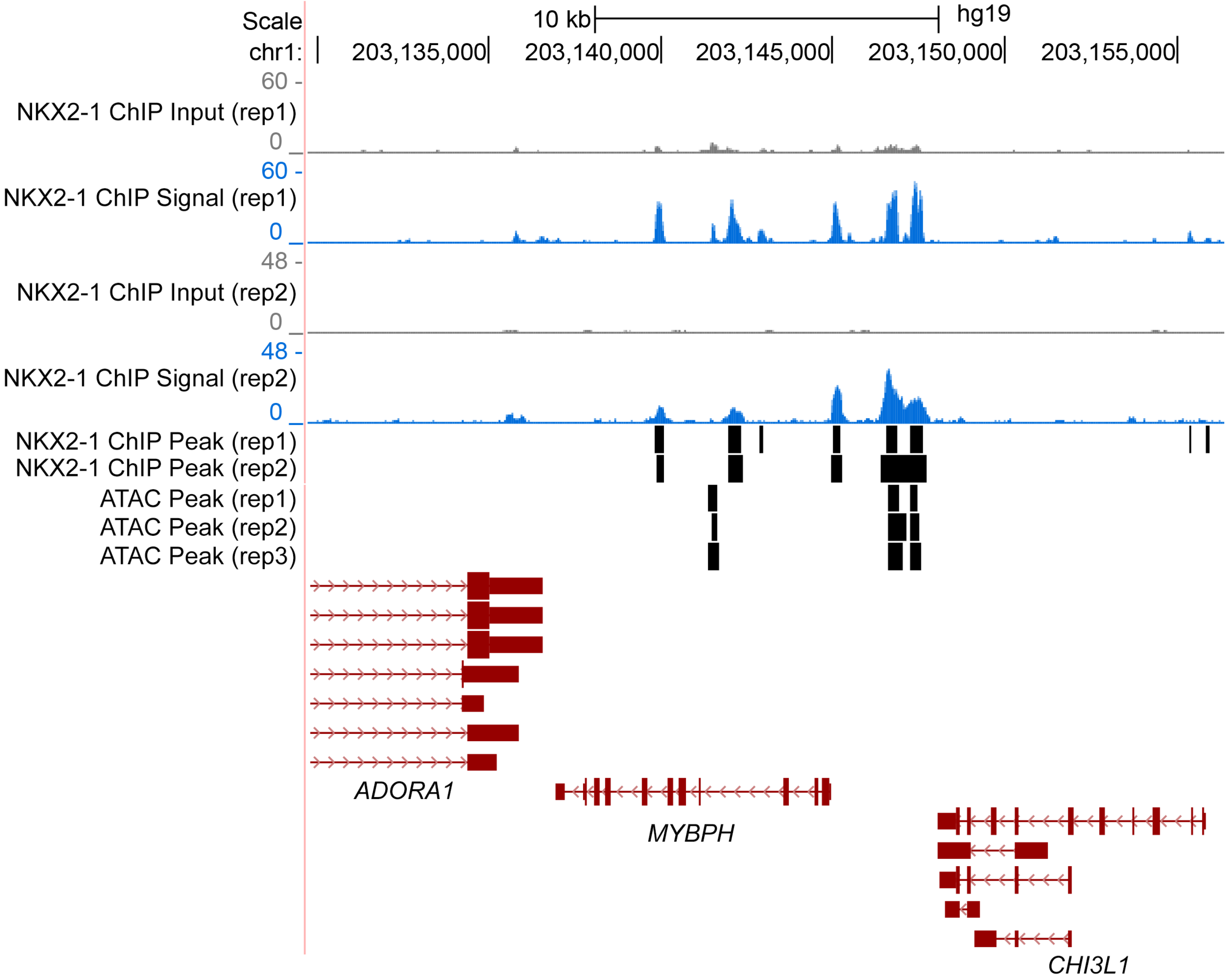

Supplementary Fig. 12. NKX2-1-binding sites at the human *MYBPH* locus were identified by two independent ChIP-seq analyses
Shown are ChIP-seq and ATAC-seq data from two or three independent experiments along with different isoforms of *MYBPH* as described in Fig. 6a.

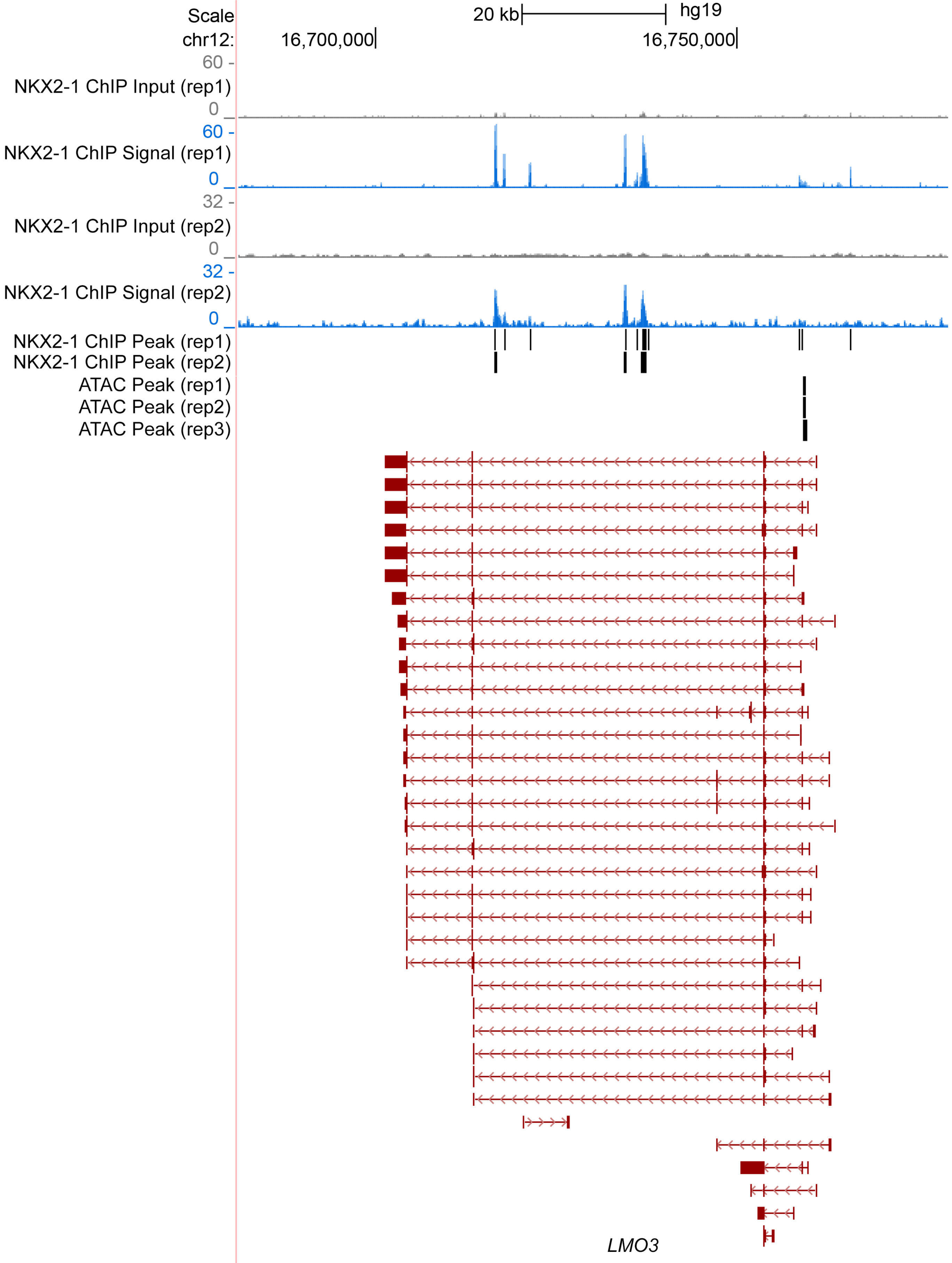

Supplementary Fig. 13. NKX2-1-binding sites at the human *LMO3* locus were identified by two independent ChIP-seq analyses
Shown are ChIP-seq and ATAC-seq data from two or three independent experiments along with different isoforms of *LMO3* as described in Fig. 7a.

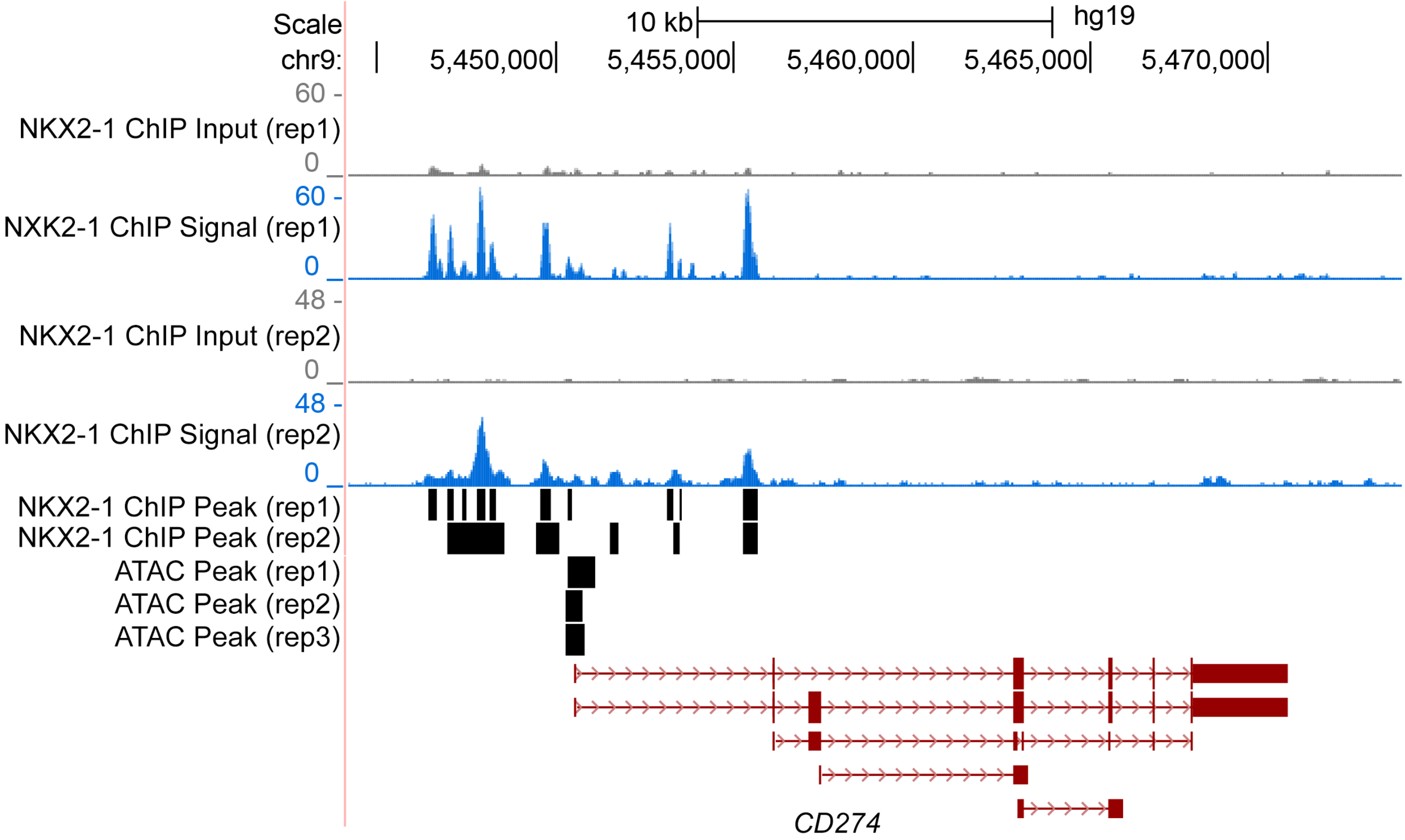

Supplementary Fig. 14. NKX2-1-binding sites at the human *CD274/PD-L1* locus were identified by two independent ChIP-seq analyses
Shown are ChIP-seq and ATAC-seq data from two or three independent experiments along with different isoforms of *CD274* as described in Fig. 8a.

Supplementary Reference

1. Stuart WD, Guo M, Fink-Baldauf IM, Coleman AM, Clancy JP, Mall MA, Lim FY, Brewington JJ, Maeda Y. CRISPRi-mediated functional analysis of lung disease-associated loci at non-coding regions, NAR Genom Bioinform. 2020 June;2(2):lqaa036. doi: 10.1093/nargab/lqaa036.
