# Supplementary material for: CRISPRi-mediated functional analysis of NKX2-1-binding sites in the lung
Source: Commun Biol. 2021 May 12;4:568. doi: 10.1038/s42003-021-02083-4 (PMC8115294; doi:10.1038/s42003-021-02083-4)
Supplement: Supplementary file 2 — Description of Additional Supplementary Files [file 42003_2021_2083_MOESM2_ESM.pdf]

Description of additional supplementary files

File name: Supplementary Data 1 – 9

Description: Supplementary datasets associated with this article. Each dataset is a separate worksheet within the Excel file.

Supplementary Data 1: Overlaps of NKX2-1 ChIP-seq peaks and/or ATAC-seq peaks in A549 lung epithelial cells

Supplementary Data 2: Results of DESeq2 differential expression analysis (Non-targted synthetic sgRNA vs. synthetic sgRNA targeting first intron of *SFTPB* [#2])

Supplementary Data 3: CRISPOR-mediated predicted matches sorted by cutting frequency determination off-target score for *SFTPB* locus

Supplementary Data 4: CRISPOR-mediated predicted matches sorted by cutting frequency determination off-target score for *LAMP3* locus

Supplementary Data 5: CRISPOR-mediated predicted matches sorted by cutting frequency determination off-target score for *SFTPA* locus

Supplementary Data 6: CRISPOR-mediated predicted matches sorted by cutting frequency determination off-target score for *MYBPH* locus

Supplementary Data 7: CRISPOR-mediated predicted matches sorted by cutting frequency determination off-target score for *LMO3* locus

Supplementary Data 8: CRISPOR-mediated predicted matches sorted by cutting frequency determination off-target score for *CD274/PD-L1* locus

Supplementary Data 9: Raw numbers and exact P values to generate bar graphs
