# Supplementary material for: CRISPRi-mediated functional analysis of NKX2-1-binding sites in the lung
Source: Commun Biol. 2021 May 12;4:568. doi: 10.1038/s42003-021-02083-4 (PMC8115294; doi:10.1038/s42003-021-02083-4)
Supplement: Supplementary file 4 — Reporting Summary [file 42003_2021_2083_MOESM4_ESM.pdf]

nature research

Corresponding author(s): YUTAKA MAEDA

Last updated by author(s): Mar 19, 2021

Reporting Summary

Nature Research wishes to improve the reproducibility of the work that we publish. This form provides structure for consistency and transparency in reporting. For further information on Nature Research policies, see our Editorial Policies and the Editorial Policy Checklist.

Statistics

For all statistical analyses, confirm that the following items are present in the figure legend, table legend, main text, or Methods section.

n/a	Confirmed	
[ ]	[x]	The exact sample size (n) for each experimental group/condition, given as a discrete number and unit of measurement
[ ]	[x]	A statement on whether measurements were taken from distinct samples or whether the same sample was measured repeatedly
[ ]	[x]	The statistical test(s) used AND whether they are one- or two-sided<br>*Only common tests should be described solely by name; describe more complex techniques in the Methods section.*
[x]	[ ]	A description of all covariates tested
[x]	[ ]	A description of any assumptions or corrections, such as tests of normality and adjustment for multiple comparisons
[ ]	[x]	A full description of the statistical parameters including central tendency (e.g. means) or other basic estimates (e.g. regression coefficient) AND variation (e.g. standard deviation) or associated estimates of uncertainty (e.g. confidence intervals)
[ ]	[x]	For null hypothesis testing, the test statistic (e.g. F, t, r) with confidence intervals, effect sizes, degrees of freedom and P value noted<br>*Give P values as exact values whenever suitable.*
[x]	[ ]	For Bayesian analysis, information on the choice of priors and Markov chain Monte Carlo settings
[x]	[ ]	For hierarchical and complex designs, identification of the appropriate level for tests and full reporting of outcomes
[x]	[ ]	Estimates of effect sizes (e.g. Cohen's d, Pearson's r), indicating how they were calculated

Our web collection on statistics for biologists contains articles on many of the points above.

Software and code

Policy information about availability of computer code

Data collection	ChIP-seq data was collected for our previous study and publicly deposited (please see 10.15252/emmm.201606711). ATAC-seq and Hi-C data were collected from ENCODE database. RNA-seq data was collected using next-generation sequencing, which is documented in Methods and a public repository site (Gene Expression Omnibus) under accession codes (GSE162186).
Data analysis	ChIP-seq data was analyzed in our previous study (please see 10.15252/emmm.201606711). ATAC-seq, RNA-seq and Hi-C data analyses are described in Methods. GraphPad Prism 8 was used for display and statistical analysis of qPCR data.

For manuscripts utilizing custom algorithms or software that are central to the research but not yet described in published literature, software must be made available to editors and reviewers. We strongly encourage code deposition in a community repository (e.g. GitHub). See the Nature Research guidelines for submitting code & software for further information.

Data

Policy information about availability of data

All manuscripts must include a data availability statement. This statement should provide the following information, where applicable:

- Accession codes, unique identifiers, or web links for publicly available datasets
- A list of figures that have associated raw data
- A description of any restrictions on data availability

A list of figures that have associated raw data is available.

Field-specific reporting

Please select the one below that is the best fit for your research. If you are not sure, read the appropriate sections before making your selection.

- [x] Life sciences
- [ ] Behavioural & social sciences
- [ ] Ecological, evolutionary & environmental sciences

For a reference copy of the document with all sections, see nature.com/documents/nr-reporting-summary-flat.pdf

Life sciences study design

All studies must disclose on these points even when the disclosure is negative.

Sample size	Biological triplicates were used in qPCR, ATAC-seq and RNA-seq experiments. Biological duplicates were used in ChIP-seq experiments. Four biological replicates were used in Hi-C experiments.
Data exclusions	No data were excluded.
Replication	All gene expression experiments were independently replicated three times.
Randomization	This is not relevant to our study since our study investigated NKX2-1 specific binding sites in the genome.
Blinding	The person who transfected synthetic sgRNA is different from the one who conducted TaqMan gene expression analysis. The person who conducted TaqMan gene expression analysis did the analysis without knowing the ID of the sgRNAs.

Reporting for specific materials, systems and methods

We require information from authors about some types of materials, experimental systems and methods used in many studies. Here, indicate whether each material, system or method listed is relevant to your study. If you are not sure if a list item applies to your research, read the appropriate section before selecting a response.

Materials & experimental systems

n/a	Involved in the study	
☐	☒	Antibodies
☐	☒	Eukaryotic cell lines
☒	☐	Palaeontology and archaeology
☒	☐	Animals and other organisms
☒	☐	Human research participants
☒	☐	Clinical data
☒	☐	Dual use research of concern

Methods

n/a	Involved in the study	
☐	☒	ChIP-seq
☒	☐	Flow cytometry
☒	☐	MRI-based neuroimaging

Antibodies

Antibodies used	All antibodies used in this study are described in Methods.
Validation	All of the antibody manufacturer's names are also described in Methods. The validation information is available from their websites.

Eukaryotic cell lines

Policy information about cell lines

Cell line source(s)	A549 and H441 cells were obtained from ATCC.
Authentication	Batch F-10600 for A549. H441 was authenticated by Genetica DNA Laboratories that is accredited by AABB.
Mycoplasma contamination	Tested negative
Commonly misidentified lines (See ICLAC register)	A549 and H441 are not commonly misidentified cell lines.

ChIP-seq

Data deposition

- [x] Confirm that both raw and final processed data have been deposited in a public database such as GEO.
- [x] Confirm that you have deposited or provided access to graph files (e.g. BED files) for the called peaks.

Data access links<br>*May remain private before publication.*	GSE86959
Files in database submission	See 10.15252/emmm.201606711
Genome browser session<br>(e.g. UCSC)	Do not have saved session

Methodology

Replicates	Two independent replicates
Sequencing depth	See 10.15252/emmm.201606711
Antibodies	See 10.15252/emmm.201606711
Peak calling parameters	See 10.15252/emmm.201606711
Data quality	See 10.15252/emmm.201606711
Software	See 10.15252/emmm.201606711
